# Prolyl-4-hydroxylase 3 maintains β cell glucose metabolism during fatty acid excess in mice

**DOI:** 10.1172/jci.insight.140288

**Published:** 2021-08-23

**Authors:** Daniela Nasteska, Federica Cuozzo, Katrina Viloria, Elspeth M. Johnson, Alpesh Thakker, Rula Bany Bakar, Rebecca L. Westbrook, Jonathan P. Barlow, Monica Hoang, Jamie W. Joseph, Gareth G. Lavery, Ildem Akerman, James Cantley, Leanne Hodson, Daniel A. Tennant, David J. Hodson

**Affiliations:** 1 Institute of Metabolism and Systems Research, University of Birmingham, Birmingham, United Kingdom.; 2 Centre for Endocrinology, Diabetes and Metabolism, Birmingham Health Partners, Birmingham, United Kingdom.; 3 Centre of Membrane Proteins and Receptors (COMPARE), University of Birmingham, Birmingham, United Kingdom.; 4 Oxford Centre for Diabetes, Endocrinology and Metabolism, University of Oxford, Oxford, United Kingdom.; 5 NIHR Oxford Biomedical Research Centre, Churchill Hospital, Oxford, United Kingdom.; 6 Department of Physiology, Anatomy and Genetics, University of Oxford, Oxford, United Kingdom.; 7 Mitochondrial Profiling Centre, School of Sport, Exercise and Rehabilitation Sciences, University of Birmingham, Birmingham, United Kingdom.; 8 School of Pharmacy, University of Waterloo, Kitchener, Ontario, Canada.; 9 Division of Systems Medicine, School of Medicine, University of Dundee, Dundee, United Kingdom.

**Keywords:** Endocrinology, Metabolism, Beta cells, Bioenergetics, Insulin

## Abstract

The **α**-ketoglutarate–dependent dioxygenase, prolyl-4-hydroxylase 3 (PHD3), is an HIF target that uses molecular oxygen to hydroxylate peptidyl prolyl residues. Although PHD3 has been reported to influence cancer cell metabolism and liver insulin sensitivity, relatively little is known about the effects of this highly conserved enzyme in insulin-secreting **β** cells in vivo. Here, we show that the deletion of PHD3 specifically in **β** cells (**β**PHD3KO) was associated with impaired glucose homeostasis in mice fed a high-fat diet. In the early stages of dietary fat excess, **β**PHD3KO islets energetically rewired, leading to defects in the management of pyruvate fate and a shift from glycolysis to increased fatty acid oxidation (FAO). However, under more prolonged metabolic stress, this switch to preferential FAO in **β**PHD3KO islets was associated with impaired glucose-stimulated ATP/ADP rises, Ca^2+^ fluxes, and insulin secretion. Thus, PHD3 might be a pivotal component of the **β** cell glucose metabolism machinery in mice by suppressing the use of fatty acids as a primary fuel source during the early phases of metabolic stress.

## Graphical abstract

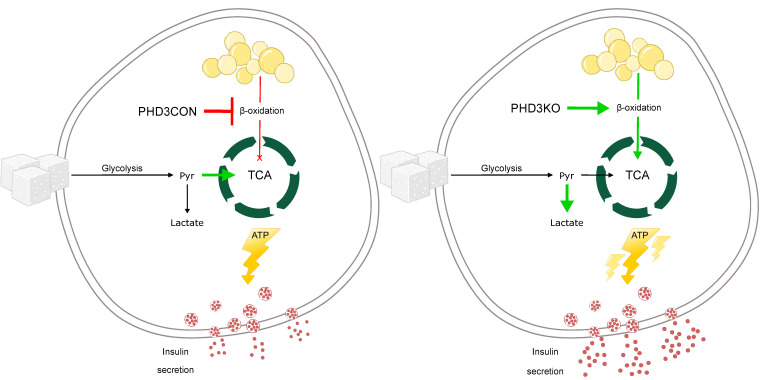

## Introduction

The prolyl-hydroxylase domain proteins (PHD1–3) encoded for by the Egl-9 homolog genes are α-ketoglutarate–dependent dioxygenases, which regulate cell function by catalyzing hydroxylation of peptidyl prolyl residues within various substrates using molecular oxygen (1–4). There are 3 well-described mammalian isozymes, PHD1, PHD2, and PHD3, which were originally described as hydroxylating the α subunit of the transcription factor HIF under normoxia (4), thus targeting it for polyubiquitylation and proteasomal degradation. When oxygen concentration becomes limited, PHD activity decreases and HIF is stabilized, leading to dimerization with the β subunit and transcriptional regulation of target genes regulating the cellular response to hypoxia (5). Although PHDs are generally regarded to be master HIF regulators, it is becoming increasingly apparent that they target a range of other substrates influencing cell function (6–9).

PHD3 is unusual among the PHDs: it is transcriptionally regulated by HIF1 during hypoxia (10), although it does not always act to destabilize HIF1 (11, 12). A number of roles for PHD3 have been described under conditions of stress or hypoxia, including macrophage influx and neutrophil survival (13, 14), apoptosis in various cancer models (8, 15, 16), and tumor cell survival (ref. 9; reviewed in ref. 17). Due to the dependence of PHD3 on α-ketoglutarate and oxygen for its activity (18), many of these actions are likely to be mediated through alterations in cell metabolism (19). Indeed, PHD3 increases glucose uptake in cancer cells through interactions with pyruvate kinase M2 (8, 20). In tumors exhibiting mutations in succinate dehydrogenase, fumarate hydratase, and isocitrate dehydrogenase 1 and 2 (21–23), PHD3 activity is altered by aberrantly high cytosolic concentrations of succinate, fumarate, and 2-hydroxyglutarate, suggesting that inactivation of this enzyme might be involved in the cellular transformation process. PHD3 has more recently been shown to hydroxylate and activate acetyl-CoA carboxylase 2 (ACC2), defined as the fatty acid oxidation gatekeeper, thus decreasing fatty acid breakdown and restraining myeloid cell proliferation during nutrient abundance (24). Together, these studies place PHD3 as a central player in the regulation of glucose and fatty acid utilization with clear implications for metabolic disease risk.

Along these lines, PHD3 has been reported to influence insulin sensitivity in the liver (25, 26), as well as maintaining glucose-stimulated insulin secretion in a rat β cell line (27). However, little is known about how PHD3 might contribute to glucose homeostasis and diabetes risk through effects directly in primary pancreatic β cells. To ensure the appropriate release of insulin, β cells have become well adapted as glucose sensors. Thus, glucose enters the β cell by facilitated diffusion through low-affinity glucose transporters (28), before conversion into glucose-6-phosphate by glucokinase and subsequent splitting into pyruvate (29). The pyruvate then undergoes oxidative metabolism in the mitochondrial matrix through the TCA cycle, driving increases in ATP/ADP ratio and leading to closure of ATP-sensitive K^+^ channels (30). This cascade triggers membrane depolarization, opening of voltage-dependent Ca^2+^ channels, influx of Ca^2+^, and Ca^2+^-dependent exocytosis of insulin vesicles through interactions with the SNARE machinery (30). Together with repression of hexokinase, monocarboxylic acid transporter 1, and lactate dehydrogenase A (31, 32), stimulus-secretion coupling prevents the inappropriate release of insulin in response to low glucose, amino acids, or lactate.

Given its reported roles in dictating fuel preference, we hypothesized that PHD3 might function as a pivotal component of the β cell glucose-sensing machinery by suppressing the use of fatty acids as an energy source (27). To further investigate PHD3-regulated β cell function in depth, we subjected a model of β cell–specific *Egln3/*PHD3 deletion to extensive in vivo and in vitro characterization, including detailed stable isotope-resolved metabolic tracing. Here, we show that the loss of PHD3 causes metabolic remodeling in the early stages of metabolic stress by shifting β cell fuel source from glucose to fatty acids. However, as metabolic stress becomes more prolonged, this energetic rewiring impairs glucose-dependent ATP/ADP ratios, Ca^2+^ fluxes, and insulin secretion.

As such, these studies build upon previous findings on PHD1–3 in islets and β cells (27) and show that PHD3 likely constitutes a fundamental mechanism to restrain fatty acid utilization and maintain glucose sensing in β cells during early stages of metabolic stress.

## Results

### Confirmation of β cell–specific PHD3 knockout.

**Figure 1:**
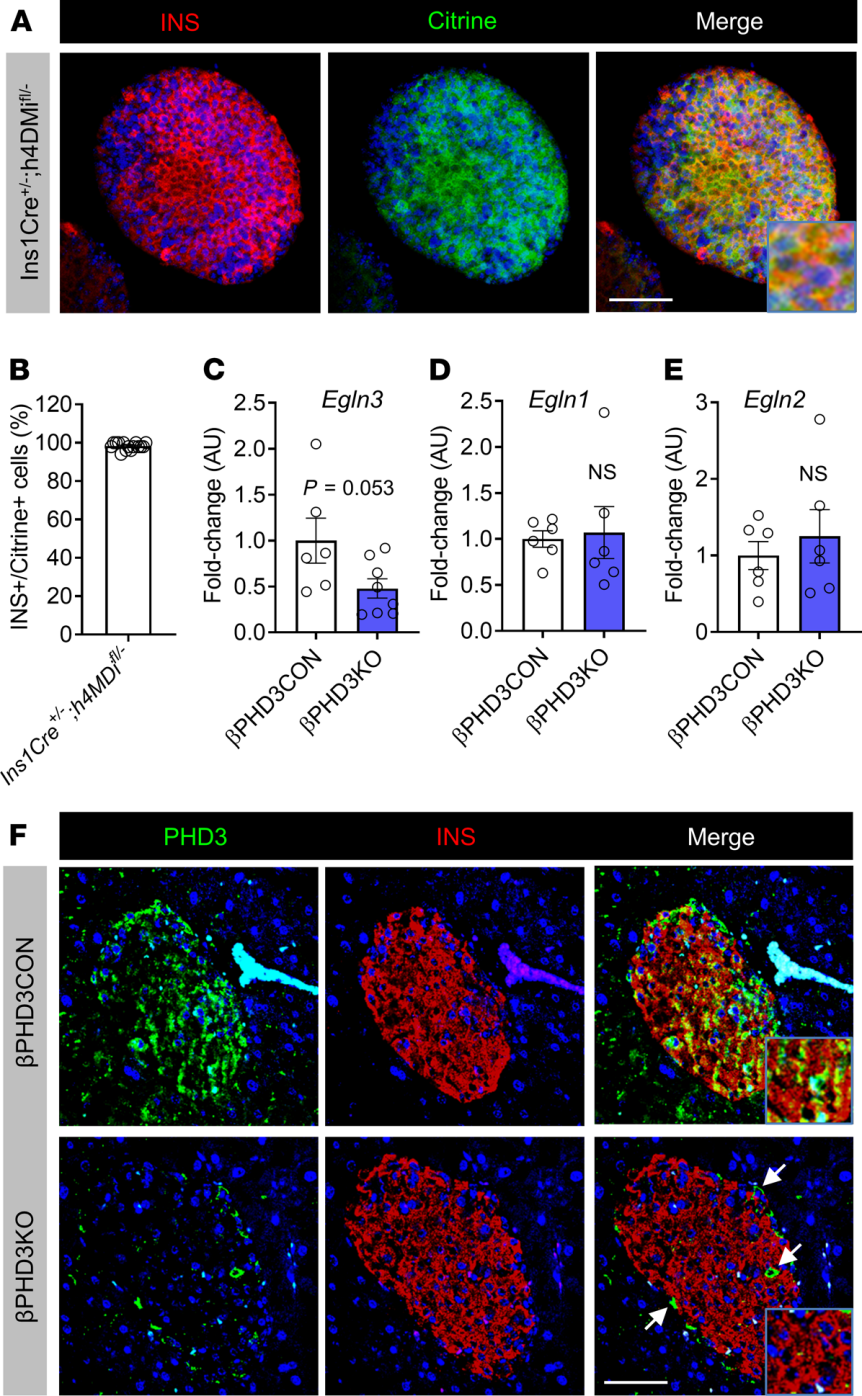
Generation and validation of mice lacking PHD3 in pancreatic β cells. (**A**) Confirmation of recombination efficiency in *Ins1Cre* islets using *R26-LSL*-*hM4Di/mCitrine* mice expressing an mCitrine reporter (representative image shown, scale bar: 42.5 μm; inset is 3.5× original magnification). (**B**) Percentage of insulin-positive (INS^+^) cells expressing mCitrine (i.e., recombined) in Ins1Cre^+/–^ h4MDi^fl/–^ islets (*n* = 15 islets). (**C**) *Egln3* expression is reduced in islets of βPHD3KO mice vs. βPHD3CON littermates (*n* = 6–8 animals/genotype, unpaired *t* test). (**D** and **E**) *Egln1* (**D**) and *Egln2* (**E**) expression levels are similar in βPHD3CON and βPHD3KO islets (*n* = 6 animals/genotype, unpaired *t* test). (**F**) PHD3 is detected in the β cell compartment of βPHD3CON but not βPHD3KO islets. Arrows show PHD3 expression in non–β cells (representative images shown, scale bar: 42.5 μm; inset is 1.75× original magnification) (*n* = 3 animals/genotype). Data shown as mean ± SEM. *Egln1/Egln2/Egln3*, Egl-9 homolog 1–3 genes; PHD3, prolyl-4-hydroxylase 3.

We first generated a model of β cell PHD3 knockout (βPHD3KO) by crossing the *Ins1Cre* deleter strain (33) with animals harboring floxed alleles for *Egln3* (34), which encodes PHD3. Given recently reported issues with allele silencing in some *Ins1Cre* colonies (35), we quantified recombination efficiency of our line using *R26-LSL*-*hM4Di/mCitrine* animals harboring an mCitrine reporter. Immunostaining of *Ins1Cre^+/–^ h4MDi^fl/–^* islets showed *Ins1Cre*-mediated recombination of the floxed allele in almost all insulin-immunopositive cells (98.3% ± 1.8%, mean ± SD; Figure 1, A and B), similar to that reported previously by us and others (33, 36, 37). As expected from this, gene expression analyses showed a 2-fold reduction in *Egln3* expression (Figure 1C), the remainder most likely reflecting the relatively higher levels of *Egln3* detected in α cells, as shown by RNA-Seq (38, 39). The loss of *Egln3* in βPHD3KO islets was not associated with compensatory changes in the other Egln paralogs, *Egln1* and *Egln2* (Figure 1, D and E). IHC analyses showed that whereas PHD3 expression was present throughout control (PHD3CON) islets, it was completely absent from β cells in βPHD3KO mice (Figure 1F). Although *Egln3* is expressed at low abundance in sorted β cells (38, 39), this is likely to be a result of profound reoxygenation after dissociation, thus suppressing *Egln3* expression (40). Together, these data show that PHD3 is expressed in β cells and can be conditionally deleted from this compartment in βPHD3KO animals, thus confirming the validity of the model.

### PHD3 does not contribute to glucose homeostasis under standard diet.

**Figure 2:**
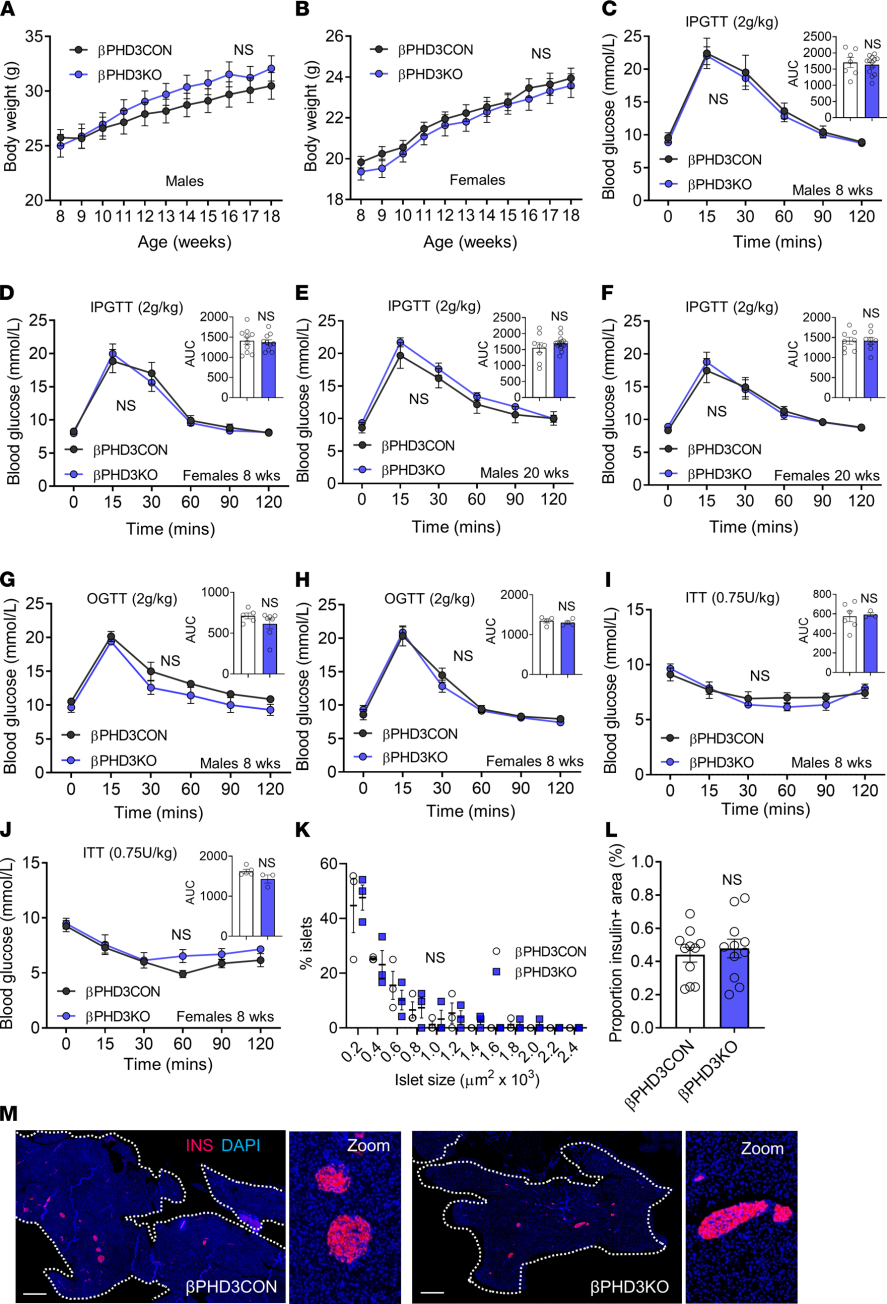
βPHD3KO in vivo phenotype under standard chow conditions. (**A** and **B**) Male (**A**) and female (**B**) βPHD3CON and βPHD3KO mice possess similar adult body weight (*n* = 8–10 male and 15 female animals/genotype, 2-way RM ANOVA; Sidak’s test). (**C** and **D**) No differences in glucose tolerance and AUC are detected between βPHD3CON and βPHD3KO male (**C**) (*n* = 13 animals/genotype) and female (**D**) (*n* = 10 animals/genotype) 8-week-old mice (2-way RM ANOVA, Sidak’s test; AUC: unpaired *t* test). (**E** and **F**) No differences in glucose tolerance and AUC during IPGTT are detected between βPHD3CON and βPHD3KO male (**E**) and female (**F**) 20-week-old mice (*n* = 8–16 male and 8 female animals/genotype; 2-way RM ANOVA, Sidak’s test; AUC: unpaired *t* test). (**G** and **H**) Oral glucose tolerance and AUC are also unchanged in βPHD3KO vs. βPHD3CON male (**G**) and female (**H**) 8-week-old mice (*n* = 3–5 male and 4 female animals/genotype; 2-way RM ANOVA, Sidak’s test; AUC: unpaired *t* test). (**I** and **J**) Insulin sensitivity and AUC are similar in βPHD3CON and βPHD3KO male (**I**) and female (**J**) 8-week-old mice (*n* = 6–7 male and 4–7 female animals/genotype; 2-way RM ANOVA, Sidak’s test; AUC: unpaired *t* test). (**K**–**M**) Cell resolution reconstruction of entire pancreatic sections shows no differences in islet size and β cell mass between βPHD3CON and βPHD3KO mice. Quantification is shown (**K** and **L**), with representative images in **M** (scale bar: 530 μm; 6.5× zoom showing maintenance of cellular resolution in a single image), **K** (*n* = 3 animals/genotype, 2-way ANOVA; Sidak’s test), and **L** (*n* = 3 animals/genotype, unpaired *t* test). Data shown are mean ± SEM. IPGTT, i.p. glucose tolerance test; OGTT, oral glucose tolerance test; ITT, insulin tolerance test.

After confirming *Egln3*/PHD3 deletion, we set out to understand the metabolic phenotype of βPHD3KO mice. βPHD3KO mice presented with normal growth curves from 8 to 18 weeks of age compared with βPHD3CON littermates, with no apparent differences in male and female cohorts (Figure 2, A and B). Intraperitoneal glucose tolerance testing (IPGTT) in the same animals showed no abnormalities in glycemia (Figure 2, C and D), which was unchanged up until the age of 20 weeks (Figure 2, E and F). Likewise, oral glucose tolerance, largely determined by incretin release from the intestine (41), was similar in βPHD3CON and βPHD3KO mice (Figure 2, G and H). As expected from the growth rates and glucose tolerance, both male and female βPHD3KO mice displayed similar insulin sensitivity to their βPHD3CON littermates (Figure 2, I and J). Finally, no differences in islet size distribution (Figure 2K) and β cell mass (Figure 2, L and M) were detected in βPHD3KO versus βPHD3CON mice.

### PHD3 does not influence β cell function in vitro under standard diet.

**Figure 3:**
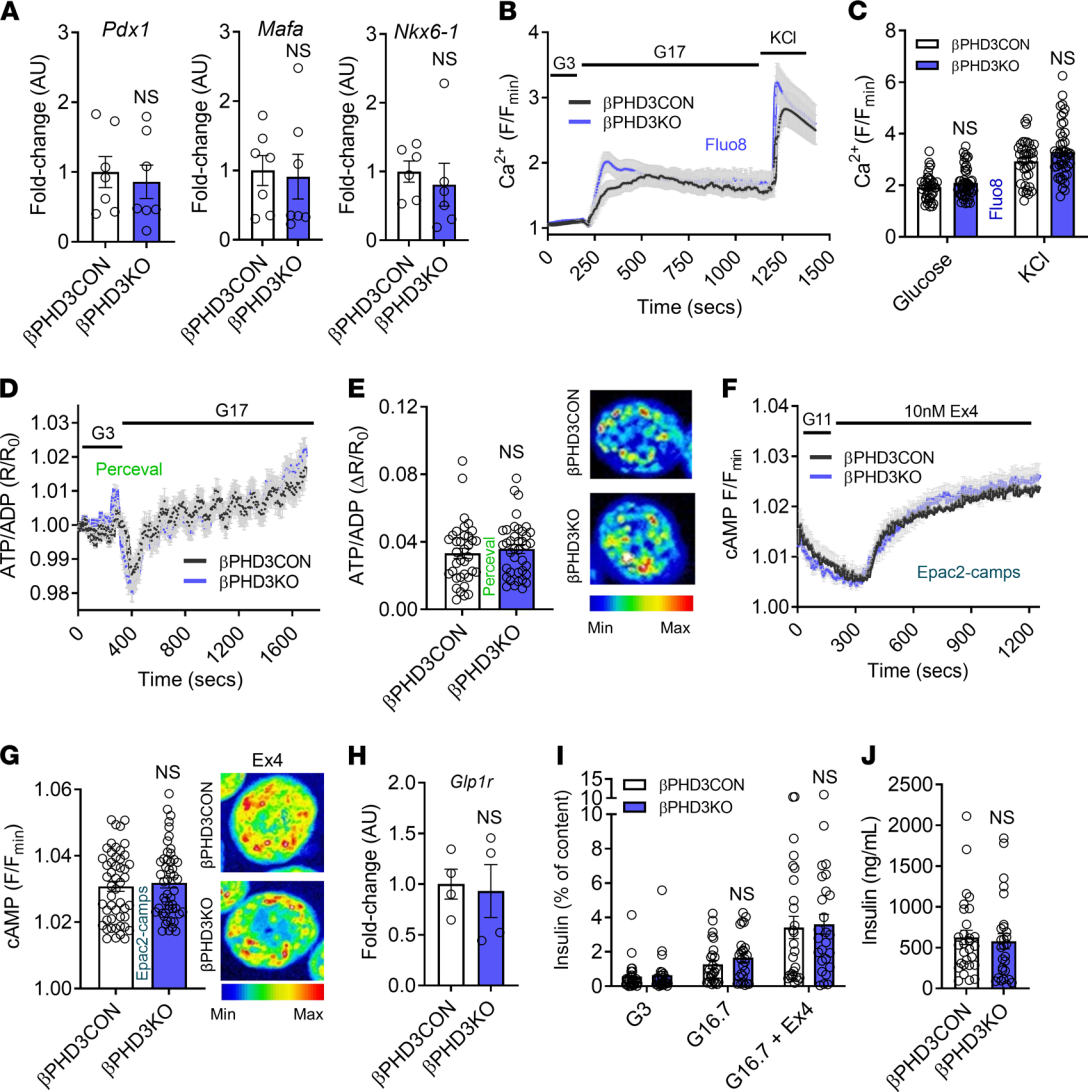
βPHD3KO in vitro phenotype under standard chow conditions. (**A**) The expression of the β cell–specific markers *Pdx1*, *Mafa*, and *Nkx6-1* is similar in βPHD3CON and βPHD3KO islets (*n* = 6–7 animals/genotype, unpaired *t* test). (**B** and **C**) Glucose- and KCl-stimulated Ca^2+^ rises do not differ in islets of βPHD3CON and βPHD3KO mice, shown by mean traces (**B**) and summary bar graph (**C**) (*n* = 38–48 islets, 4–5 animals/genotype; 2-way ANOVA, Sidak’s test). (**D** and **E**) Glucose-stimulated ATP/ADP rises are similar in βPHD3CON and βPHD3KO islets, shown by mean traces (**D**) and summary bar graph (**E**) (representative images shown; a single islet has been cropped for clarity; *n* = 36–39 islets, 4–5 animals/genotype, unpaired *t* test). (**F** and **G**) cAMP responses to Ex4 do not differ between βPHD3CON and βPHD3KO islets, shown by mean traces (**F**) and summary bar graph (**G**) (representative images shown; a single islet has been cropped for clarity; *n* = 50 islets, 4–5 animals/genotype, unpaired *t* test). (**H**) *Glp1r* expression is similar in βPHD3CON and βPHD3KO islets (*n* = 4 animals/genotype, unpaired *t* test). (**I**) Insulin secretory responses to glucose and Exendin-4 show no differences between βPHD3CON and βPHD3KO islets (*n* = 29 replicates, 6 animals/genotype, 2-way ANOVA; Sidak’s test). (**J**) Total insulin content also remained similar between βPHD3CON and βPHD3KO islets (*n* = 29 replicates, 6 animals/genotype; unpaired *t* test). Data shown as mean ± SEM. G3, 3 mM glucose; G16.7, 16.7 mM glucose; G17, 17 mM glucose.

Isolation of islets for more detailed in vitro workup revealed normal expression of the β cell transcription factors *Pdx1*, *Mafa*, and *Nkx6-1* in βPHD3KO islets, suggesting that β cell differentiation was unaffected by the loss of PHD3 (Figure 3A). Further suggestive of mature β cell function, live imaging approaches revealed normal glucose-stimulated Ca^2+^ fluxes (Figure 3, B and C) and ATP/ADP ratios (Figure 3, D and E) in βPHD3KO islets. Suggesting the presence of intact glucagon-like peptide-1 receptor (GLP1R) signaling, an important amplifying input for insulin secretion, cAMP responses to the incretin-mimetic Exendin-4 (Figure 3, F and G) as well as *Glp1r* expression (Figure 3H) were similar in βPHD3CON and βPHD3KO islets. In line with the Ca^2+^, ATP/ADP, and cAMP analyses, both glucose-stimulated and Exendin-4–potentiated insulin secretion were similar in islets isolated from male and female βPHD3CON and βPHD3KO animals (Figure 3, I and J).

### Loss of PHD3 improves insulin secretion at the onset of metabolic stress.

We next examined whether PHD3 might play a more important role in regulating insulin release during metabolic stress. Therefore, male animals were placed on a high-fat diet (HFD) to induce obesity and metabolic stress (42).

**Figure 4:**
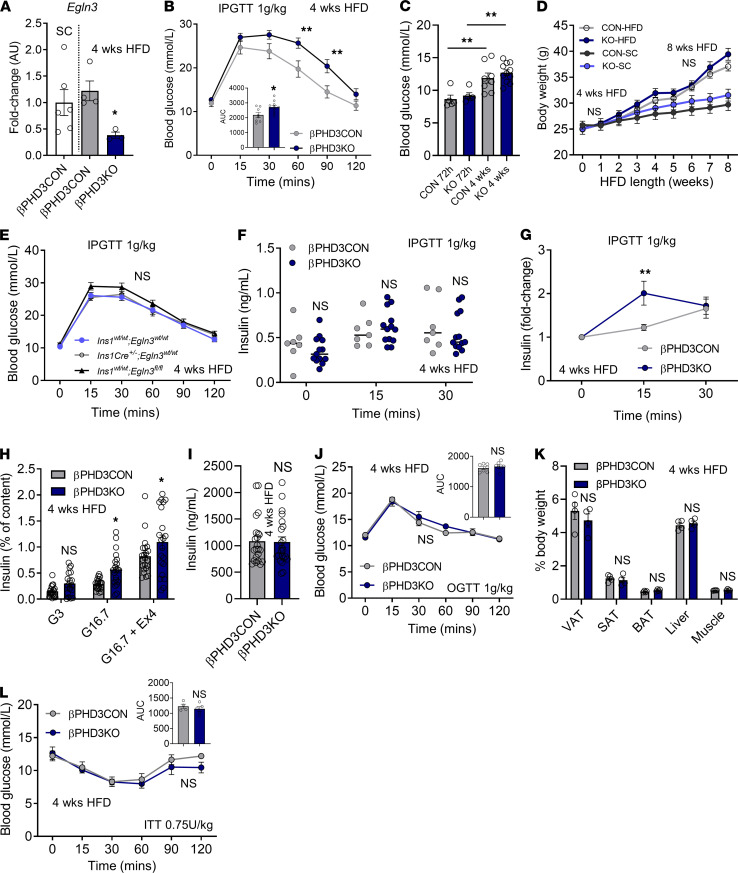
βPHD3KO in vivo and in vitro phenotype during early metabolic stress (4 weeks HFD). (**A**) *Egln3* remains suppressed in βPHD3KO islets after 4 weeks HFD (*n* = 3–6 animals/genotype; unpaired *t* test). (**B** and **C**) Glucose tolerance (**B**) is impaired in male βPHD3KO mice after 4 weeks HFD, although fasting glucose levels (**C**) are unaffected after 72 hours HFD (*n* = 8–11 animals/genotype; 2-way RM ANOVA, Sidak’s test). (**D**) Body weight is similar in male HFD-fed βPHD3CON and βPHD3KO animals (*n* = 11–12 animals/genotype; 2-way RM ANOVA, Sidak’s test). Body weight data from Figure 2A are included for comparison. (**E**) Glucose tolerance is unaffected in male *Cre-*only and *Egln3*^fl/fl^-only controls (*n* = 10–13 animals/genotype; 2-way RM ANOVA, Tukey’s test). (**F**) Serum insulin levels after glucose challenge are similar in βPHD3CON and βPHD3KO mice (*n* = 7–13 mice/genotype; 2-way RM ANOVA, Sidak’s test). (**G**) Insulin responses to glucose, shown by stimulation index, are higher in male βPHD3KO mice (*n* = 7–13 animals/genotype; 2-way RM ANOVA, Sidak’s test). (**H** and **I**) Glucose-stimulated and Exendin-4–potentiated insulin secretion are increased in βPHD3KO islets (**H**) (*n* = 20 replicates, 4 animals/genotype; 2-way ANOVA, Sidak’s test), whereas insulin content (**I**) remains unchanged (*n* = 20 replicates, 4 animals/genotype; unpaired *t* test). (**J**) βPHD3CON and βPHD3KO mice show similar oral glucose tolerance (*n* = 7 animals/genotype; 2-way RM ANOVA, Sidak’s test). (**K**) No changes in body composition are seen in βPHD3KO vs. βPHD3CON mice (*n* = 4 animals/genotype; 2-way ANOVA, Sidak’s test). (**L**) Insulin sensitivity remains unchanged in βPHD3KO mice (*n* = 4–5 animals/genotype; 2-way RM ANOVA, Sidak’s test). Data shown as mean ± SEM. *P < 0.05, **P < 0.01, and NS. VAT/SAT/BAT, visceral/subcutaneous/brown adipose tissue; SC, standard chow; HFD, high-fat diet; IPGTT, i.p. glucose tolerance test.

After 4 weeks HFD, *Egln3* was moderately upregulated in βPHD3CON islets (Figure 4A), although this was not significant. However, *Egln3* levels remained suppressed in βPHD3KO islets (Figure 4A). GTT revealed significantly impaired glucose homeostasis in βPHD3KO mice after 4 weeks HFD feeding but not after 72 hours HFD feeding (Figure 4, B and C), despite similar body weight gain compared with βPHD3CON littermates (Figure 4D). The 72-hour time point was used to differentiate the effects of early and prolonged fatty acid incorporation/utilization. As expected, fasting blood glucose levels were elevated in βPHD3CON mice after 4 weeks HFD (Figure 4C). There was no effect of *Cre* or floxed alleles per se on the metabolic phenotype after 4 weeks HFD, with *Ins1^wt/wt^ Egln3^fl/fl^*, *Ins1Cre^+/–^ Egln3^wt/wt^*, and *Ins1^wt/wt^ Egln3^wt/wt^* controls being indistinguishable (Figure 4E). After 4 weeks HFD, IPGTT showed no difference in the serum insulin levels between βPHD3CON and βPHD3KO mice under fasting and glucose-stimulated conditions (Figure 4F). However, βPHD3KO mice mounted earlier and larger magnitude insulin secretory responses to glucose bolus, as shown by the stimulation index (Figure 4G). Islets isolated from the same animals secreted significantly more insulin in glucose-stimulated and Exendin-4–potentiated states (Figure 4H), and insulin content was similar to βPHD3CON littermates (Figure 4I). Finally, 4 weeks of HFD feeding had no effect on glucose tolerance during oral GTT (OGTT; Figure 4J), body composition (Figure 4K), and insulin sensitivity (Figure 4L) in βPHD3KO mice versus βPHD3CON littermates.

Thus, βPHD3KO mice were glucose intolerant on an HFD, showed improved insulin secretion, and were able to release a greater fraction of their insulin granules (i.e., were more sensitized to exocytosis). These data raise the possibility that nutrient-sensing and utilization might have been altered in βPHD3KO islets.

### PHD3 maintains glycolysis and pyruvate management in β cells.

**Figure 5:**
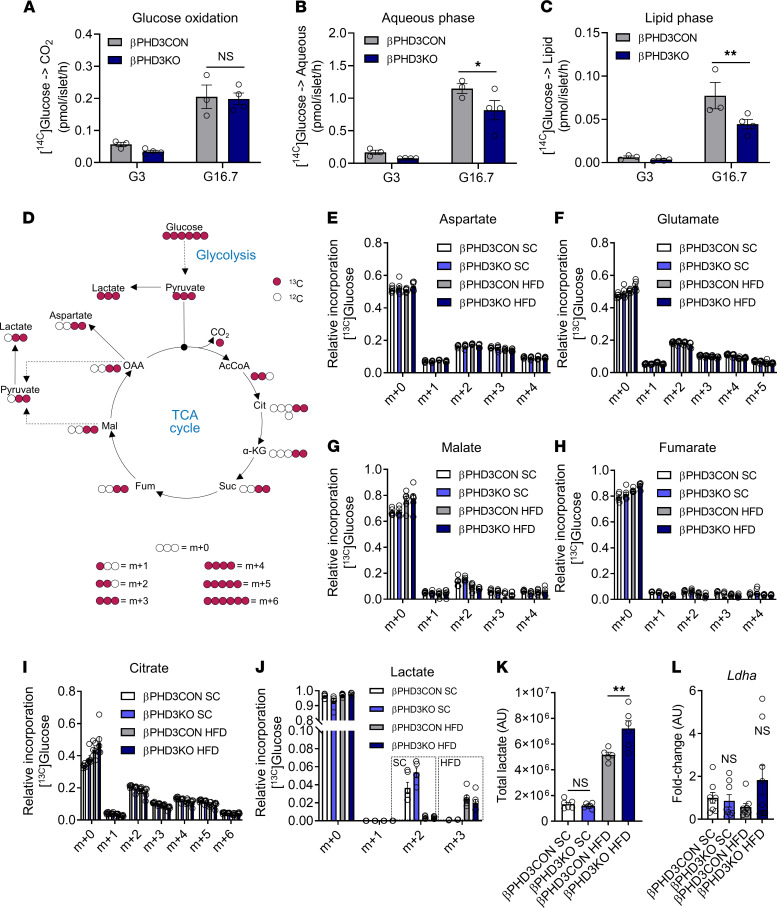
Metabolic rewiring in βPHD3KO islets during metabolic stress. (**A**–**C**) βPHD3KO islets possess intact glucose oxidation (**A**) but have an impaired accumulation of glycolytic/TCA cycle metabolites (**B**) and glucose-driven lipogenesis (**C**) after 4 weeks of HFD (*n* = 3 islet preparations, 3 animals/genotype; 2-way ANOVA, Benjamini-Krieger-Yekutieli 2-stage procedure). (**D**) Schematic showing ^13^C from ^13^C_6_-[U]-glucose incorporation into metabolites in βPHD3CON and βPHD3KO islets. (**E**–**I**) Mass isotopomer distributions (MID) showing that ^13^C incorporation from glucose into aspartate (**E**), glutamate (**F**), malate (**G**), fumarate (**H**), or citrate (**I**) is similar in SC and HFD βPHD3CON and βPHD3KO islets from animals on either SC or 4 weeks HFD (*n* = 6 islet preparations, 3 animals/genotype, 2-way ANOVA, Tukey’s test). (**J**) ^13^C from ^13^C_6_-[U]-glucose is incorporated primarily into m+2 lactate in SC βPHD3CON and βPHD3KO islets, whereas a shift to m+3 lactate is seen during 4 weeks HFD feeding (*n* = 6 islet preparations, 3 animals/genotype; 2-way ANOVA, Tukey’s test). (**K**) Steady-state lactate levels are increased in βPHD3KO vs. βPHD3CON islets after 4 weeks HFD (*n* = 6 islet preparations, *n* = 3 animals/genotype; 1-way ANOVA, Sidak’s test). (**L**) *Ldha* expression is not significantly different in βPHD3CON and βPHD3KO islets from animals on either SC or HFD (*n* = 8–9 animals/genotype; Dunnett’s test). Data shown as mean ± SEM. *P < 0.05, **P < 0.01, and NS. SC, standard chow; HFD, high-fat diet.

Given the reported roles of PHD3 in glycolysis, we wondered whether the changes in β cell function observed during the early phases of HFD feeding in βPHD3KO mice might be associated with changes in glucose metabolism. We first looked at glycolytic fluxes using ^14^C glucose. Although glucose oxidation was not altered at low or high glucose in islets from βPHD3KO mice after 4 weeks HFD feeding (Figure 5A), there was a small but significant decrease in ^14^C content in the aqueous phase, indicating a net reduction in TCA cycle/other metabolites derived from glycolysis (Figure 5B). Notably, a 2-fold reduction in the incorporation of glucose into the lipid pool (i.e., glucose-driven lipogenesis) was also detected in βPHD3KO islets after 4 weeks HFD (Figure 5C), suggestive of a decreased oxidative pyruvate entry into the TCA cycle and lipogenic acetyl-CoA (43).

To gain a higher resolution analysis of glucose fate, stable isotope-resolved tracing was performed in βPHD3KO islets using ^13^C_6_-[U]-glucose. The schematic in Figure 5D depicts the fate of ^13^C from ^13^C_6_-[U]-glucose in βPHD3CON and βPHD3KO islets, as assessed by gas chromatography–mass spectrometry (GC-MS). Analysis of mass isotopomer distribution showed no differences in ^13^C incorporation into aspartate, glutamate, malate, fumarate, or citrate in βPHD3CON and βPHD3KO islets on either standard chow (SC) or 4 weeks HFD (Figure 5, E–I). Thus, although the contribution of glucose to aqueous cellular metabolite pools was clearly reduced in βPHD3KO islets after 4 weeks HFD (Figure 5B), there was no net change in the incorporation of ^13^C from glucose into each metabolite, i.e., the TCA cycle proceeded normally despite lowered glucose fluxes. Islets from animals fed SC showed m+2 lactate accumulation (Figure 5J), which is consistent with lactate normally produced as a result of oxidative metabolism of glucose-derived pyruvate. However, during HFD there was a pronounced switch to reduction of pyruvate to lactate (indicated by the m+3 isotopomer) in both genotypes (Figure 5J).

Further analysis of steady-state lactate levels showed a significant increase in lactate production in islets from HFD-fed βPHD3KO versus βPHD3CON mice (Figure 5K). Together with the m+2 → m+3 switch, this finding supports initial measures with ^14^C glucose indicating reduced fueling of the TCA cycle by glycolysis (Figure 5B). Furthermore, the tracing data suggest that βPHD3KO islets after 4 weeks HFD increased the reduction of pyruvate → lactate to support continued glycolysis through regeneration of the cytosolic NAD^+^ pool. Although the expression of the “disallowed gene” *Ldha* (31, 32) tended to be increased, this was variable and not significantly different between βPHD3CON and βPHD3KO islets (Figure 5L).

Together, these data suggest that metabolic stress induced defects in the management of pyruvate fate in βPHD3KO islets, implying that insulin secretion in vitro must be maintained and even amplified through mechanisms other than glycolysis.

### PHD3 suppresses fatty acid use under metabolic stress.

**Figure 6:**
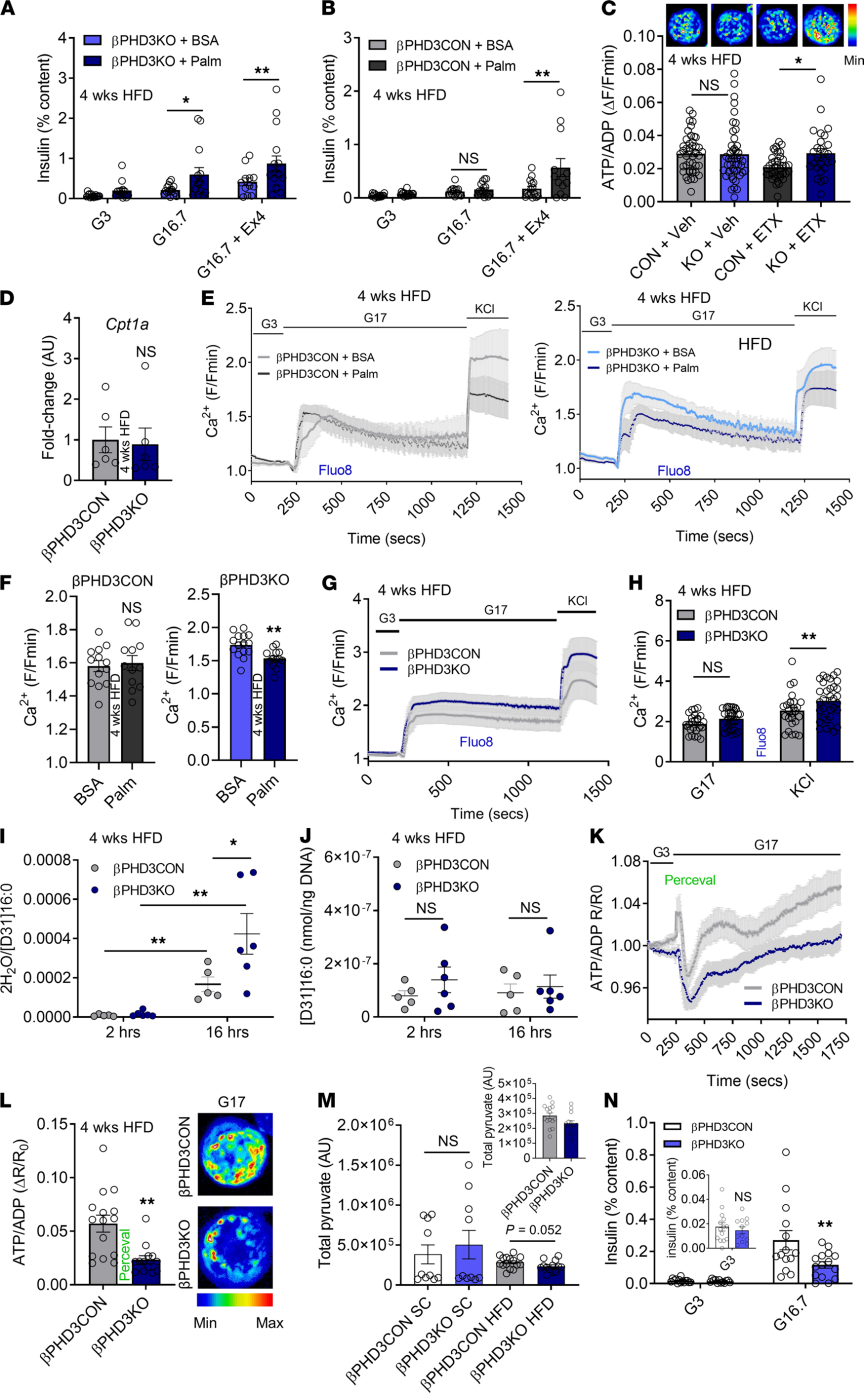
Nutrient preference is altered in βPHD3KO islets during early metabolic stress (4 weeks HFD). (**A**) Palmitate (Palm) enhances glucose-stimulated and Exendin-4–stimulated insulin secretion in βPHD3KO islets (*n* = 12–17 replicates, 7–9 animals/genotype; 2-way ANOVA, Benjamini-Krieger-Yekutieli 2-stage procedure). (**B**) Same as for **A** but showing glucose and Exendin-4 response in βPHD3CON islets (*n* = 13–17 replicates, 7–9 animals/genotype; 2-way ANOVA, Benjamini- Krieger-Yekutieli 2-stage procedure). (**C**) Etomoxir (ETX) increases glucose-stimulated ATP/ADP ratio in βPHD3KO islets (representative images show a single islet; *n* = 27–45 islets, 5–6 animals/genotype; 2-way ANOVA, Sidak’s test). (**D**) *Cpt1a* expression is similar in βPHD3CON and βPHD3KO islets (*n* = 6 animals/genotype; unpaired *t* test). (**E** and **F**) Palmitate impairs Ca^2+^ responses to glucose in βPHD3KO islets, shown by mean traces (**E**) and bar graphs (**F**) (*n* = 13–15 islets, 2–3 animals/genotype, unpaired *t* test). (**G** and **H**) Glucose-stimulated and KCl-stimulated Ca^2+^ rises are similar to controls (glucose), or increased (KCl), in βPHD3KO islets, shown by mean traces (**G**) and a bar graph (**H**) (*n* = 26–33 islets, 6 animals/genotype; 2-way ANOVA, Sidak’s test). (**I**) The 2H_2_O/D31-palmitate ratio is increased in βPHD3KO islets after 16 hours (*n* = 5–6 animals/genotype; within genotype: unpaired *t* test; between genotype: 2-way ANOVA, Sidak’s test). (**J**) D31-palmitate tracer uptake is similar in βPHD3CON and βPHD3KO islets (*n* = 5–6 animals/genotype; 2-way ANOVA, Sidak’s test). (**K** and **L**) ATP/ADP rises are impaired in βPHD3KO islets, shown by mean traces (**K**) and bar graph and representative images (**L**) (single islet shown; *n* = 13–15 islets, 4 animals/genotype, unpaired *t* test). (**M**) Steady-state pyruvate levels are decreased in βPHD3KO islets (*n* = 11–13 replicates, 5–8 animals/genotype; Mann-Whitney *U* test). (**N**) Low glucose preincubation decreases glucose-stimulated insulin secretion in βPHD3KO islets from animals on SC (*n* = 14–15 replicates, 6 animals/genotype; 2-way ANOVA, Sidak’s test). Data shown are mean ± SEM. *P < 0.05, **P < 0.01, and NS. SC, standard chow; HFD, high-fat diet.

We hypothesized that βPHD3KO islets might switch to an alternative energy source to sustain their function, namely, β-oxidation of fatty acids, which are present in excess during HFD feeding. Moreover, in cancer cells, PHD3 has been shown to increase activity of ACC2, which converts acetyl-CoA → malonyl-CoA, the latter suppressing carnitine palmitoyltransferase I (CPT1), the rate-limiting step in fatty acid oxidation (24, 44). Indicating a profound change in β cell nutrient preference, supplementation of the culture medium with the fatty acid palmitate for 48–72 hours augmented glucose-stimulated and Extendin-4–potentiated insulin secretion in βPHD3KO islets after 4 weeks HFD (Figure 6A). By contrast, βPHD3CON islets after 4 weeks HFD showed no changes in glucose-stimulated insulin release after culture with palmitate (Figure 6B), confirming that the fatty acid was unlikely to induce lipotoxicity at the concentration and timing used here. Interestingly, 48–72 hours incubation with palmitate increased Extendin-4–potentiated insulin secretion in βPHD3CON islets after 4 weeks HFD (Figure 6B).

Providing evidence for increased CPT1 activity in βPHD3KO islets after 4 weeks HFD, the CPT1a inhibitor etomoxir was able to augment ATP/ADP responses to glucose in these islets (Figure 6C), despite unchanged mRNA levels of *Cpt1a* (Figure 6D). In line with this finding, culture with low palmitate concentration decreased glucose-stimulated Ca^2+^ fluxes in βPHD3KO but not in βPHD3CON islets after 4 weeks HFD (Figure 6, E and F), presumably due to an increased flux of fatty acid–derived acetyl-CoA into the TCA cycle. Although glucose-driven Ca^2+^ fluxes were apparently normal in βPHD3KO islets after 4 weeks HFD, this was likely due to an increased sensitivity of voltage-dependent Ca^2+^ channel to membrane depolarization, because responses to KCl were significantly elevated (Figure 6, G and H).

To gain a higher resolution view of fatty acid fate, we incubated 4 weeks HFD βPHD3CON and βPHD3KO islets with D31-palmitate, before measuring the intracellular D31-palmitate concentration and 2H_2_O released from fatty acid oxidation. With this assay, the ratio of 2H_2_O to intracellular D31-palmitate provides a measure of fatty acid oxidation while accounting for any differences between tracer uptake/turnover. Confirming the accuracy of the assay, 2H_2_O/D31-palmitate values were robustly increased after 16 hours versus 2 hours incubation with tracer (Figure 6I). Notably, after 4 weeks HFD, 2H_2_O/D31-palmitate values were significantly higher in βPHD3KO versus βPHD3CON islets at the 16-hour time point (Figure 6I), indicative of higher fatty acid oxidation rates. The uptake of tracer was similar in βPHD3KO versus βPHD3CON islets (Figure 6J).

Taken together, these data strongly suggest that PHD3 loss led to alterations in fatty acid utilization in islets.

### Loss of PHD3 decreases dependency on glucose as a fuel source.

We wondered whether increased fatty acid utilization in βPHD3KO islets after 4 weeks HFD was associated with a decreased dependency on glucose as a primary fuel source. Confirming a switch away from glycolysis, glucose-stimulated ATP/ADP ratios were markedly decreased in βPHD3KO islets after 4 weeks HFD (Figure 6, K and L), despite the apparent increases in insulin secretion (Figure 6A). Moreover, steady-state pyruvate levels were decreased in βPHD3KO islets after 4 weeks HFD (Figure 6M). Last, glucose-stimulated insulin secretion (GSIS) was impaired in SC βPHD3KO islets that were starved of glucose (3 mM) for 3 hours prior to challenge (Figure 6N), presumably due to dysregulated use of alternative fuel sources, which then inhibit critical metabolic hubs in central carbon metabolism, such as pyruvate dehydrogenase. These data confirm the presence of defective pyruvate handling and suggest that βPHD3KO islets alter pyruvate production and/or increase pyruvate → lactate conversion to maintain redox balance during HFD.

Thus, after 4 weeks HFD, βPHD3KO islets became less reliant on glycolysis to fuel ATP/ADP production, were able to sustain oxidative phosphorylation through fatty acid use, and secreted more insulin when both glucose and fatty acids were present.

### Regulated gene expression of ACC1 and ACC2 in β cells.

**Figure 7:**
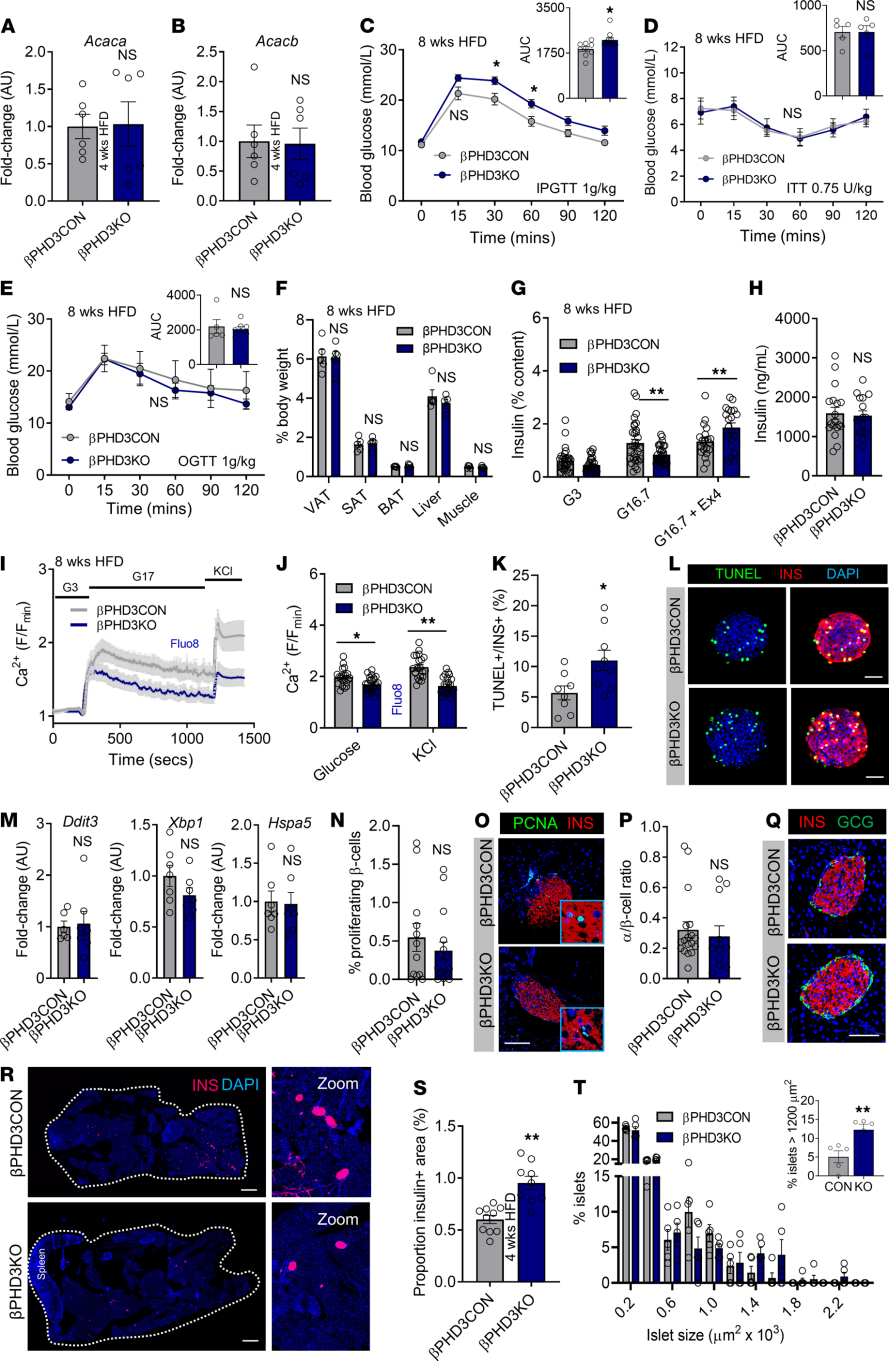
Prolonged metabolic stress (8 weeks HFD) leads to insulin secretory failure in βPHD3KO islets. (**A** and **B**) *Acaca* (**A**) and *Acacb* (**B**) expression is similar in βPHD3CON and βPHD3KO islets from animals fed 4 weeks HFD (*n* = 6 animals/genotype; unpaired *t* test). (**C**) Glucose tolerance remains impaired in βPHD3KO mice after 8 weeks HFD feeding (*n* = 9–11 animals/genotype; 2-way RM ANOVA, Sidak’s test; AUC: unpaired *t* test). (**D**) Insulin sensitivity is unchanged in βPHD3KO mice (*n* = 5 animals/genotype, 2-way RM ANOVA; Sidak’s test; AUC: unpaired *t* test). (**E**) Oral glucose tolerance is normal in βPHD3KO mice (*n* = 6–7 animals/genotype; 2-way RM ANOVA, Sidak’s test; AUC: unpaired *t* test). (**F**) Body composition is unchanged in βPHD3KO mice (*n* = 5 animals/genotype; 2-way ANOVA, Sidak’s test). (**G** and **H**) Glucose-stimulated insulin secretion (**G**) is impaired in βPHD3KO islets after 8 weeks HFD (*n* = 29–32 replicates, 4 animals/genotype; 2-way ANOVA, Sidak’s test), despite similar insulin content (**H**) (16–18 replicates, 4 animals/genotype; unpaired *t* test). (**I** and **J**) Glucose-stimulated and KCl-stimulated Ca^2+^ rises are impaired in βPHD3KO islets, shown by mean traces (**I**) and quantification (**J**) (*n* = 21–24 islets/genotype, 2 animals/genotype; 2-way ANOVA, Sidak’s test). (**K** and **L**) Apoptosis is increased in βPHD3KO islets, shown by quantification (**K**) and representative images (**L**) (*n* = 8–9 islets/genotype; unpaired *t* test). (**M**) *Ddit3*, *Xbp1*, and *Hspa5* expression shows no changes in βPHD3KO islets (*n* = 6–7 animals/genotype; unpaired *t* test). (**N**–**Q**) Islet proliferation (PCNA; **N** and **O**) and α cell/β cell ratio (**P** and **Q**) are unchanged in βPHD3KO islets (*n* = 11–18 islets, 3–4 animals/genotype; unpaired *t* test). (**R**–**T**) Images (**R**) and quantification (**S** and **T**) showing increased β cell mass in βPHD3KO mice (scale bar: 530 μm; inset is 5.25× original magnification) (*n* = 3 animals/genotype, 2-way ANOVA; unpaired *t* test). Data shown are mean ± SEM. *P < 0.05, **P < 0.01, and NS. Scale bar: 42.5 μm (inset 4× original magnification) unless otherwise stated. PCNA, proliferating cell nuclear antigen; SC, standard chow; HFD, high-fat diet.

Previous studies have shown that PHD3 maintains glucose metabolism by hydroxylating and activating ACC2 (encoded by *Acacb*), which inhibits CPT1 through generation of mitochondrial malonyl-CoA, thus suppressing use of fatty acids via β-oxidation (45, 46). However, β cells are thought to predominantly express ACC1 (encoded by *Acaca*; refs. 45, 46), which supplies cytosolic malonyl-CoA to fatty acid synthase for de novo lipid biosynthesis rather than for β-oxidation (43). Therefore, we sought to determine whether it was possible for PHD3 to act via ACC2 in pancreatic β cells. We reexamined the expression of *ACACB* in pancreatic β cells in multiple well-powered human bulk islet and purified β cell gene expression data sets (38, 47, 48). *ACACB* mRNA was found to be present in β cells, albeit at much lower levels than *ACACA* mRNA (Supplemental Figure 1A). Our data suggest that the presence of *ACACB* mRNA in β cells was not artifactual: first, the mRNA levels of *ACACB* were comparable to the β cell transcription factor *HNF1A*, suggesting ample gene expression levels compatible with function (Supplemental Figure 1A). Second, the *ACACB* gene promoter was bound by islet and β cell–specific transcription factors, suggesting that *ACACB* is a bona fide β cell gene under the regulation of cell-specific transcription factors (Supplemental Figure 1B). Our findings thus suggest that as long as protein translation occurs, PHD3 could have maintained glucose metabolism in pancreatic β cells via hydroxylation of ACC2. We next examined if *ACACB* gene expression is under the regulation of PHD3 protein. Gene expression levels of *Acaca* and *Acacb* were similar in βPHD3CON and βPHD3KO islets after 4 weeks HFD (Figure 7, A and B), suggesting that *Acacb* mRNA levels were not regulated by PHD3 activity.

Thus, *ACACB* is present in β cells, and contains promoter regions regulated by β cell–specific transcription factors, but does not depend upon PHD3 for expression. These data are consistent with a scenario whereby PHD3 hydroxylates ACC2 without influencing mRNA expression.

### PHD3 protects against insulin secretory failure during prolonged metabolic stress.

Last, we sought to understand the phenotype of βPHD3KO mice when faced with continued fatty acid/nutrient abundance. Glucose intolerance was still present in βPHD3KO mice after 8 weeks on an HFD (Figure 7C) although less severe than after 4 weeks HFD, suggesting that metabolic rewiring might, in fact, have been protective against prolonged exposure to excess fatty acids in vivo. As was the case after 4 weeks HFD, βPHD3KO mice showed similar insulin sensitivity to βPHD3CON after 8 weeks HFD (Figure 7D). In contrast to the IPGTT data, oral glucose tolerance was preserved at this time point in βPHD3KO mice, suggesting an intact incretin action (Figure 7E). Furthermore, after 8 weeks HFD, the body composition of βPHD3KO mice was similar to βPHD3CON (Figure 7F). By this point, however, impaired glucose-stimulated insulin secretion (Figure 7G) was apparent in isolated βPHD3KO islets. This secretory deficit could be rescued by application of Extendin-4 to sensitize insulin granules to exocytosis (Figure 7, G and H), as expected from the oral glucose testing results. In addition, after 8 weeks HFD, the amplitude of glucose-stimulated Ca^2+^ rises was significantly reduced in βPHD3KO compared with βPHD3CON islets (Figure 7, I and J).

Suggesting that profound defects in voltage-dependent Ca^2+^ channels might also be present, responses to the generic depolarizing stimulus KCl were markedly blunted in the same islets (Figure 7, I and J). Although apoptosis was increased in βPHD3KO islets after 8 weeks HFD (Figure 7, K and L), this did not reflect a (detectable) lipotoxic ER stress response because *Ddit3*, *Hspa5*, and *Xbp1* (Figure 7M) expression remained unchanged. Moreover, after 8 weeks HFD, proliferating cell nuclear antigen (PCNA) staining (Figure 7, N and O) and the α cell/β cell ratio (Figure 7, P and Q) were similar in βPHD3CON and βPHD3KO islets, suggesting that β cells were unlikely to be less/more proliferative or adopting α cell features (or vice versa). Nonetheless, a profound 2-fold increase in β cell mass was observed in βPHD3KO mice after 8 weeks HFD (Figure 7, R and S), with a significant increase in the proportion of larger islets (Figure 7T), implying that either apoptosis was restricted to smaller/medium islets or changes in the apoptosis/proliferation rate had not yet been able to counter previous β cell mass expansion.

### Loss of PHD3 is not associated with changes in HIF stabilization.

Previous studies have shown that PHD3 is highly regulated at the transcriptomic level by hypoxia (10), and in line with this, we also found that *Egln3* levels in WT islets were increased under hypoxic (1% O_2_) conditions (Supplemental Figure 2A). To account for HIF-dependent effects on β cell phenotype in SC βPHD3KO animals, a number of canonical HIF1α target genes were assessed. Notably, the levels of *Bnip3*, *Car9*, and *Gls* were similar between normoxic (21% O_2_) SC βPHD3CON and βPHD3KO islets (Supplemental Figure 2, B–D). Further suggesting the presence of intact HIF signaling, *Bnip3* and *Car9* were upregulated to similar levels in hypoxic (1% O_2_) SC βPHD3CON and βPHD3KO islets, whereas *Gls* did not reliably increase (Supplemental Figure 2, B–D). Glucose and KCl-stimulated Ca^2+^ fluxes, shown to be sensitive to HIF stabilization (49), were similar in βPHD3CON and βPHD3KO islets exposed to hypoxia (Supplemental Figure 2, E–H).

Suggesting that the stabilization of HIF1α and HIF2α was unlikely to be a major feature in 4 weeks HFD βPHD3KO islets, *Bnip3*, *Car9*, and *Gls* levels were similar to βPHD3CON (Supplemental Figure 2, I–K). Furthermore, after 8 weeks HFD, the HIF2α target *Ccnd1* remained similar in βPHD3CON and βPHD3KO islets, whereas the gene *Dll4* was downregulated (Supplemental Figure 2, L and M).

## Discussion

**Figure 8:**
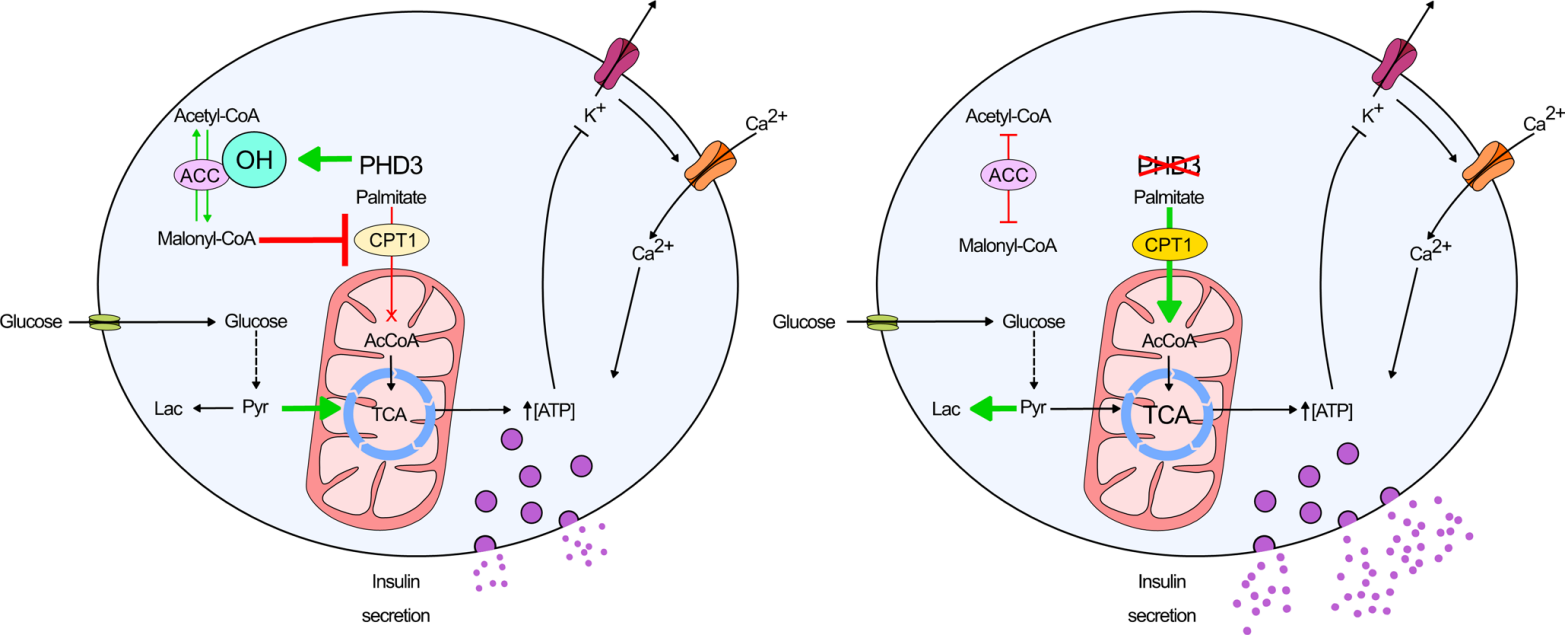
Schematic showing the proposed changes that occur in βPHD3KO islets after HFD feeding. In βPHD3CON islets, glucose is converted to pyruvate, before entering the TCA cycle to drive ATP production and insulin secretion. PHD3 activity leads to generation of malonyl-CoA, which inhibits CPT1 to suppress oxidation of fatty acids. By contrast, in βPHD3KO islets, CPT1 is no longer inhibited, allowing β-oxidation of fatty acids to proceed. As a result, fatty acid–derived acetyl-CoA feeds the TCA cycle and generates ATP/ADP while glycolytically derived pyruvate is converted to lactate to maintain REDOX status. PHD3, prolyl-4-hydroxylase 3; CPT1, carnitine palmitoyltransferase I.

In the present study, we show that the α-ketoglutarate–dependent PHD3 maintained β cell glucose sensing under states of metabolic stress associated with fatty acid abundance. Our data suggest that PHD3 is required for ensuring that acetyl-CoA derived from glycolysis preferentially feeds the TCA cycle, linking blood glucose levels with ATP/ADP generation, β cell electrical activity, and insulin secretion. The loss of PHD3 led to metabolic remodeling under HFD, resulting in decreased glycolytic fluxes, an increase in lactate accumulation, and utilization of fatty acids as an energy source. Thus, PHD3 appears to be a critical component of the β cell metabolic machinery required for glucose sensing during episodes of nutritional overload (Figure 8).

Previous studies have shown that the PHD1–3 inhibitor ethyl-3,4-dihydroxybenzoate exerts bimodal effects on islets: low concentrations increase GSIS, whereas high doses impair GSIS (27). Suggesting that these changes are mediated primarily by PHD3, siRNAs against PHD1 and PHD2 are without effect on GSIS in INS1-832/13 clonal rat β cells, whereas PHD3 siRNA markedly blunts release of the hormone (27). Using a conditional knockout model, our studies extend these findings to primary islets and provide further mechanistic evidence for a critical role of PHD3 in β cell metabolism and function. A key difference between the studies is that PHD3 loss only impairs GSIS in islets exposed to metabolic stress (HFD), whereas the effects were apparent in INS1-832/13 under normal culture conditions. The most likely explanation for this finding is the different metabolic dependencies of primary islets versus proliferative, immortalized β cells.

How does PHD3 maintain glucose metabolism in β cells? Previous studies in cancer cells and skeletal muscle have shown that PHD3 hydroxylates and activates ACC2, suppressing β-oxidation (24). Although β cells are thought to predominantly express ACC1, the levels of *ACACB*, which encodes ACC2, were found to be similar to the β cell transcription factor HNF1A, albeit lower than those of *ACACA*. We thus propose that the loss of PHD3 might plausibly lead to suppression of ACC2 activity, which becomes apparent during HFD when its substrate is present in abundance. Alternatively, PHD3 might hydroxylate and activate ACC1, leading to regulation of CPT1 by malonyl-CoA when fatty acids are supplied in excess, as suggested by glucose oxidation experiments. In both cases, identifying the PHD3 hydroxylation sites involved will be critical. However, assigning hydroxylation targets using mass spectrometry is currently controversial: misalignment of hydroxylation is frequently associated with the presence of residues in the tryptic fragment that can be artifactually oxidized (44, 50). Thus, studies using animals lacking PHD3 and ACC1/ACC2 in β cells, or alternatively the use of (relatively) specific inhibitors, would be required to definitively link the carboxylase with the phenotype here.

As normal chow contains a low proportion of calories from fat, metabolic stress was needed to reveal the full in vitro and in vivo phenotype associated with PHD3 loss. These data also support an effect of PHD3 on ACC1/ACC2 and CPT1, because without acyl-CoA derived from exogenous fatty acids, glucose would still constitute the primary fuel source and regulator of insulin release. The lack of phenotype under normal diet is unlikely to reflect the age of the animals, because even at 20 weeks of age, glucose intolerance was still not present in βPHD3KO mice. Of interest, the severity of the βPHD3KO in vivo phenotype was milder after 8 weeks versus 4 weeks HFD feeding, despite the presence of impaired glucose-dependent β cell function by this time point. These observations suggest that by 8 weeks HFD, compensatory protective mechanisms may become upregulated as a consequence of the metabolic rewiring in β cells. It will be necessary in the future to investigate the mechanistic/phenotypic changes occurring during even longer duration HFD feeding (e.g., 12–20 weeks). It will also be interesting to understand how PHD3 activity changes in other models of metabolic stress, such as *db/db* and *ob/ob* mice.

Suggesting that the phenotype associated with PHD3 loss was not due to changes in HIF signaling, no differences in the gene expression of HIF1 targets could be detected in βPHD3KO versus βPHD3CON islets. Indeed, PHD2 is the major hydroxylase that regulates HIF1α stability (11, 12), with no changes in activity of the transcription factor after PHD3 loss (11, 12, 51). Thus, it is perhaps unsurprising that there was a lack of HIF1 transcriptional signature in βPHD3KO islets, in agreement with previous studies in other tissues (25, 51). In addition, glucose-stimulated Ca^2+^ fluxes, a sensitive readout of changes in oxygen-dependent regulation (49), were unaffected during hypoxia in βPHD3KO islets. Although there was a trend toward increased *Ldha* expression in HFD βPHD3KO islets, this was just a fraction of that previously reported in hypoxic rodent islets (52). Nonetheless, we cannot completely exclude HIF-dependent effects, and, as such, studies should be repeated either on an HIF1-null and HIF2-null background (i.e., a quadruple transgenic) or using (moderately) specific chemical inhibitors.

We acknowledge a number of limitations with the present studies. First, workup was limited to rodents, and it will be important to confirm whether or not results translate to human islets. Although our attempts at silencing PHD3 using *EGLN3* shRNA were unsuccessful, studies using (relatively) specific PHD3 inhibitors are warranted. Second, interactions between PHD3 and ACC2 are inferred from our metabolic workup and known biochemistry. Identifying hydroxylation sites and creating corresponding ACC1/ACC2 mutants is needed, but current mass spectrometry analysis is challenging due to the assignment of false positives, as mentioned above. Third, we focused our studies on 4 and 8 weeks of HFD, and it is unclear whether the switch toward increased fatty acid utilization might be maladaptive or protective in βPHD3KO mice during longer periods of HFD feeding. Fourth, HFD studies were restricted to male animals, and further studies should be extended to female animals. Although sex differences in phenotype were not observed under standard diet, we cannot exclude a sexually dimorphic effect of HFD.

In summary, PHD3 possesses a conserved role in gating nutrient preference toward glucose and glycolysis during both cell transformation (24) and metabolic stress (as shown here). It will be interesting to now study whether similar effects of PHD3 are present in other cell types involved in glucose-sensing (for example, pancreatic α cells, hypothalamic neurons).

## Methods

### Experimental design.

No data were excluded unless the cells displayed a nonphysiological state (i.e., impaired viability). All individual data points are reported. The measurement unit is animal or batch of islets, with experiments replicated independently. Animals and islets were randomly allocated to treatment groups to ensure that all states were represented in the different experiment arms.

### Mouse models.

βPHD3KO mice were generated using the Cre-*LoxP* system. *Ins1Cre* mice (JAX stock 026801), with Cre recombinase knocked into the *Ins1* gene locus, were crossbred to mice carrying floxed alleles for PHD3 (*Egln^3^^fl/fl^*; ref. 34). Adult βPHD3KO animals (*Ins1Cre^+/–^ Egln3^fl/fl^*) and their controls (βPHD3CON: *Ins1^wt/wt^ Egln3^fl/fl^*, *Ins1Cre^+/–^ Egln3^wt/wt^*, and *Ins1^wt/wt^ Egln3^wt/wt^*) were used from 8 to 20 weeks of age under both standard diet and HFD conditions. No extrapancreatic recombination has been observed in *Ins1Cre* mice, and possession of a *Cre* allele is not associated with any changes in glucose homeostasis in our hands (33, 36). Recombination efficiency of the *Ins1Cre* allele was checked using a *R26-LSL*-*hM4Di/mCitrine* (JAX stock 026219) DREADD reporter strain. Animals were maintained on a C57BL/6J background and backcrossed for at least 6 generations following rederivation into the animal facility. Lines were regularly refreshed by crossing to bought-in C57BL/6J (Charles River Laboratories). WT male CD1 mice aged 8–12 weeks (Charles River Laboratories) were used for confirmation of gene expression under hypoxic (1% O_2_) conditions. βPHD3CON and βPHD3KO mice were fed SC and/or an HFD containing 60% fat (Research Diets, catalog D12492), with body weight checked weekly until 18–20 weeks of age. Animals were maintained in a specific pathogen–free facility, with free access to food and water.

### IPGTT and OGTT.

Mice were fasted for 4 to 6 hours, before i.p. injection of glucose. Animals on SC received 2 g/kg body weight glucose, whereas those on HFD received a lower dose of 1 g/kg body weight. In our hands, this allows the measurement of blood glucose concentration without the need to dilute samples and decreases adverse reactions associated with profound hyperglycemia. Blood samples for glucose measurement were taken from the tail vein 0, 15, 30, 60, 90, and 120 minutes after the glucose challenge. Glucose was measured using a Contour XT glucometer (Bayer). For mice on SC, IPGTT was performed every 2 to 4 weeks, between 8 and 20 weeks of age. HFD-fed mice underwent IPGTT after 72 hours, 4 weeks, and 8 weeks of HFD feeding. OGTT was performed as for IPGTT, except that glucose was delivered using an oral gavage tube (2 g/kg and 1 g/kg body weight in SC-fed and HFD-fed mice, respectively).

### Serum insulin.

Blood samples were collected after i.p. glucose injection (1 g/kg body weight). Serum was separated by centrifugation, before assaying using the HTRF Mouse Serum Insulin Assay kit (Cisbio). Due to NC3R limits on blood sample volumes, insulin was measured only 0, 15, and 30 minutes after glucose injection.

### Insulin tolerance test.

Mice fasted for 4 to 6 hours (SC and 4 weeks HFD cohorts) or overnight (8 weeks HFD cohort) received 0.75 U/kg body weight insulin (Humulin S, 100 U/mL, Lilly) given by i.p. injection. Blood glucose was measured 0, 15, 30, 60, 90, and 120 minutes after insulin injection.

### Body composition measurement.

Male βPHD3CON and βPHD3KO mice fed HFD for 4 and 8 weeks were weighed and sacrificed by cervical dislocation. The following tissues were harvested and weighed immediately postmortem: visceral fat (epididymal fat pads), subcutaneous fat, brown adipose tissue, liver, and muscle (quadriceps femoris). Tissue contribution to body composition was expressed as percentage of body weight.

### Islet isolation.

Islets were isolated after bile duct injection with NB8 1 mg/mL collagenase (Serva) and Histopaque/Ficoll gradient separation (MilliporeSigma). Islets were cultured in RPMI medium containing 10% FCS, 100 U/mL penicillin, and 100 μg/mL streptomycin (MilliporeSigma) at 5% CO_2_, 37°C. For experiments under hypoxia, islets were incubated in a Don Whitely H35 Hypoxystation, allowing oxygen tension to be finely regulated at either 1% or 21%.

### Gene expression.

TRIzol (Thermo Fisher Scientific) purification was used for mRNA extraction, whereas cDNA was synthesized by reverse transcription. Gene expression was detected by quantitative real-time PCR, using PowerUp SYBR Green Master Mix (Thermo Fisher Scientific), and quantification was based on the 2^–ΔΔCt^ method, expressed as fold change in gene expression. The sequences of the forward and reverse primers used in the study can be found in Supplemental Table 1.

### IHC.

Pancreata were isolated, fixed in 10% formalin, and embedded in paraffin. Paraffin slides were deparaffinized and rehydrated, before antigen retrieval using citrate buffer. Sections stained for PHD3 were incubated overnight at 4°C with guinea pig anti-insulin 1:100 (Abcam, ab7842) and rabbit anti-PHD3 1:100 (Novus Bio, NB100-139), followed by washing and 2 hours incubation at room temperature with anti–guinea pig Alexa Fluor 568 1:300 (Thermo Fisher Scientific, A-11075) and anti-rabbit Alexa Fluor 488 1:1000 (Thermo Fisher Scientific, A-21206). PCNA staining was carried out using rabbit anti-insulin 1:500 (Cell Signaling Technology, 3014S) and mouse anti-PCNA 1:500 (Cell Signaling Technology, 2586) as primary antibodies. Secondary antibodies used were anti-rabbit Alexa Fluor 568 1:500 (Thermo Fisher Scientific, A-10042) and anti-mouse Alexa Fluor 488 (Thermo Fisher Scientific; A11001). VECTASHIELD HardSet mounting medium with DAPI (Vector Laboratories) was used to mount coverslips on the sections.

Images were taken using a Zeiss LSM780 meta-confocal microscope equipped with highly sensitive GaAsP PMT detectors. Excitation was delivered at λ = 405 nm, λ = 488 nm, and λ = 561 nm for DAPI, Alexa Fluor 488, and Alexa Fluor 568, respectively. For PHD3 staining, the emitted signals were detected at λ = 410 to 472 nm, λ = 507 to 596 nm, and λ = 570 to 694 nm, for DAPI, Alexa Fluor 488, and Alexa Fluor 568, respectively. For PCNA staining, emitted signals were detected at λ = 418 to 507 nm, λ = 507 to 552 nm, and λ = 579 to 641 nm for DAPI, Alexa Fluor 488, and Alexa Fluor 568, respectively.

TUNEL staining was performed using the DeadEnd Fluorometric TUNEL System (Promega), as previously described (53). The proportion of apoptotic β cells was calculated as the area of TUNEL^+^ staining (fluorescein-12-dUTP)/area of insulin^+^ staining (as described above). The α cell/β cell ratio was calculated after staining with rabbit antibodies against insulin (as described above) and glucagon (primary antibody: mouse anti-glucagon 1:2000, MilliporeSigma, G2645; secondary antibody goat anti-mouse Alexa Fluor 488, 1:500, Thermo Fisher Scientific, A11001). Images were captured as described above. Excitation was delivered at λ = 405 nm, λ = 488 nm, and λ = 633 nm for DAPI, fluorescein-12-dUTP/Alexa Fluor 488, and Alexa Fluor 647, respectively. Emitted signals were detected at λ = 428 to 533 nm, λ = 498 to 559 nm, and λ = 643 to 735 nm for DAPI, fluorescein-12-dUTP/Alexa Fluor 488, and Alexa Fluor 633, respectively. For β cell mass analysis, sections were incubated with rabbit anti-insulin 1:500 (Cell Signaling Technology, 3014S) and mouse anti-glucagon 1:2000 (MilliporeSigma, G2654), followed by washing and application of goat anti-rabbit Alexa Fluor 647 1:500 (Thermo Fisher Scientific, A-21244) and goat anti-mouse DyLight 488 1:500 (Invitrogen, Thermo Fisher Scientific, 35503). Coverslips were mounted using VECTASHIELD HardSet with DAPI and 425 images per section captured using a Zeiss Axio Scan.Z1 automated slide scanner equipped with a 20×/0.8 NA objective. β Cell mass (%) was calculated as the area of insulin^+^ staining/area of the pancreas. Excitation was delivered at λ = 330 to 375 nm and λ = 590 to 650 nm for DAPI and Alexa Fluor 647, respectively. Emitted signals were detected using an Orca Flash 4.0 at λ = 430 to 470 nm and λ = 663 to 738 nm for DAPI and Alexa Fluor 647, respectively.

### Insulin secretion in vitro and insulin measurement.

Islets (10 to 15 size matched) were stimulated with 3 mM glucose, 16.7 mM glucose, and 16.7 mM glucose plus 20 nM Exendin-4 in HEPES-bicarbonate buffer (mM: 120 NaCl, 4.8 KCl, 24 NaHCO_3_, 0.5 Na_2_HPO_4_, 5 HEPES, 2.5 CaCl_2_, 1.2 MgCl_2_; MilliporeSigma) supplemented with 0.1% BSA at 37°C. Insulin content was extracted using acid ethanol. Insulin concentration (ng/mL) was measured using an HTRF Insulin Ultra-Sensitive Assay kit (PerkinElmer, 62IN2PEG). For experiments with exogenous lipids, islets were treated with either 0.75% BSA control or 150 μM sodium palmitate dissolved in 0.75% BSA for 48 to 72 hours before the secretion assay. This concentration and timing do not induce profound lipotoxicity in our hands, allowing the study of metabolic phenotype in the absence of β cell failure.

### Live imaging.

Islets were loaded with the Ca^2+^ indicators Fluo8 (AAT Bioquest, 21083) or Fura2 (AAT Bioquest, 21020), before imaging using a Crest X-Light spinning disk microscope coupled to a Nikon Ti-E base with 10 × 0.4 NA and 20 × 0.8 NA objectives. For Fluo8 imaging, excitation was delivered at λ = 458 to 482 nm using a Lumencor Spectra X light engine. Emission was captured at λ = 500 to 550 nm using a highly sensitive Photometrics Delta Evolve EM-CCD. For experiments with the ratiometric Ca^2+^ indicator, Fura2, excitation was delivered at λ = 340 nm and λ = 385 nm using Cairn Research Fura LEDs in widefield mode, with emitted signals detected at λ = 470 to 550 nm.

For ATP/ADP imaging, islets were transduced with the ATP/ADP sensor Perceval (a gift from Gary Yellen, Harvard University, Boston, Massachusetts, USA; ref. 54) using an adenoviral vector and imaged identically to Fluo8. For FRET-based cAMP imaging, islets were infected with adenovirus harboring Epac2-camps (a gift from Dermot Cooper, University of Cambridge, Cambridge, United Kingdom). Excitation was delivered at 430 to 450 nm, with emission detected at λ = 460 to 500 and λ = 520 to 550 nm for Cerulean and Citrine, respectively.

In all cases, HEPES-bicarbonate buffer was used (mM: 120 NaCl, 4.8 KCl, 24 NaHCO_3_, 0.5 Na_2_HPO_4_, 5 HEPES, 2.5 CaCl_2_, 1.2 MgCl_2_, and 3–17 d-glucose), with glucose and drugs (Exendin-4, MilliporeSigma E144-.1MG, and etomoxir, MilliporeSigma E1905-5MG) being added at the indicated concentrations and time points. Fura2 and Epac2-camps traces were normalized as the ratio of 340:385 or Cerulean/Citrine, respectively. Data were presented as raw or F/F_min_ where F = fluorescence at any time point and F_min_ = minimum fluorescence, or R/R_0_ where R = fluorescence at any time point and R_0_ = fluorescence at 0 minutes.

### Glucose oxidation assays and metabolic tracing.

For ^14^C glucose oxidation and lipid incorporation, batches of 40 islets were used for quantification of ^14^C glucose (PerkinElmer) oxidation and incorporation into lipids by scintillation spectrometry, as previously described (43).

GC-MS–based ^13^C_6_ mass isotopomer distribution was assessed as follows. To ensure steady state, 50 to 100 islets were cultured with 10 mM ^13^C_6_-[U]-glucose (MilliporeSigma, 389374) for 24 hours (55), before extraction of metabolites using sequentially prechilled HPLC-grade methanol, HPLC-grade distilled H_2_O containing 1 μg/mL D6-glutaric acid, and HPLC-grade chloroform at –20°C (all from MilliporeSigma). Polar fractions were separated by centrifugation, vacuum dried, and solubilized in 2% methoxyamine hydrochloric acid in pyridine (Thermo Fisher Scientific). Samples were derivatized using *N*-tertbutyldimethylsilyl-*N*-methyltrifluoroacetamide with 1% (w/v) tertbutyldimethyl-chlorosilane (both from MilliporeSigma), before analysis on an Agilent 7890B gas chromatograph mass spectrometer, equipped with a medium polar range polydimethylsiloxane GC column (DB35-MS). Mass isotopomer distributions were determined based upon spectra corrected for natural isotope abundance. Data were analyzed using MetaboliteDetector software (56).

### D31-palmitate incorporation and oxidation assays.

For D31-palmitate tracing, 140 islets per genotype were cultured at 5% CO_2_, 37°C, in a solution of 150 μM D31-palmitic acid (98%; Cambridge Isotope Laboratories, DLM-215-1), and dissolved in RPMI supplemented with 10% FBS, 100 U/mL penicillin, 100 μg/mL streptomycin, and 10% BSA. At 2 hours and 16 hours after incubation, 70 islets per genotype were collected in 250 μL of PBS and lysed prior to DNA quantification and freezing at –20°C. A 200 μL aliquot of D31-palmitate–labeled solution was also collected and stored at –20°C. Similarly, upon overnight incubation, the remaining islets were collected in PBS and lysed and the DNA was quantified. The leftover labeling solution was also collected and frozen at –20°C for measures of background signal.

Total lipids were extracted from cell lysates (57) and prepared and analyzed by a 6890N Network GC System (Agilent Technologies) as previously described (58). An internal standard containing a known concentration was added to samples for the quantification of total fatty acids. Fatty acid methyl esters were identified by their retention times compared with a standard containing 31 known fatty acids. Intracellular D31 enrichment was determined by GC-MS using a 5890 GC coupled to a 5973N MSD (Agilent Technologies). Ions with mass-to-charge ratios (*m/z*) of M+0 and M+31 were determined by selected ion monitoring. As a marker of fatty acid oxidation, we measured the appearance of 2H_2_O derived from D31-palmitate in cell media using a Finnigan GasBench-II (Thermo Fisher Scientific; ref. 59).

### Visualization of transcriptomic data sets.

Details of the RNA-Seq and ChIP-Seq experiments, as well as human islet donors, were previously described (48, 60–62). All transcriptomic data sets used to generate Supplemental Figure 1, A and B, are publicly available through EMBL-EBI and GEO databases and freely accessible through http://pasqualilab.upf.edu/app/isletregulome For visualization, processed RNA-Seq and ChIP-Seq (bigWig) data files were downloaded (EBI: E-MTAB-1919, E-MTAB-1294 and GEO: GSE151405) and loaded onto the local open source University of California Santa Cruz Genome Browser (http://genome.ucsc.edu/; ref. 63), under a private session.

### Statistics.

Measurements were performed on discrete samples unless otherwise stated. Data normality was assessed using D’Agostino-Person test. All analyses were conducted using GraphPad Prism software. Pairwise comparisons were made using Student’s 2-tailed unpaired or paired *t* test. Multiple interactions were determined using 1-way ANOVA or 2-way ANOVA, adjusted for repeated measures where relevant. Pairwise post hoc testing was performed using Sidak’s test, or Tukey’s test where more than 2 groups were considered. Where a highly significant interaction was detected using 2-way ANOVA, but post hoc testing was just above *P* = 0.05, multiple comparisons were accounted for using the FDR followed by the 2-stage linear step-up method of Benjamini, Krieger, and Yekutieli. For nonparametric multiple comparison, Kruskal-Wallis test was used followed by Dunn’s post hoc test. Degrees of freedom were accounted for during all post hoc testing. A *P* value of less than 0.05 was considered significant.

### Study approval.

Animal studies were regulated by the Animals (Scientific Procedures) Act 1986 of the United Kingdom (Personal Project Licence P2ABC3A83), and approval was granted by the University of Birmingham’s Animal Welfare and Ethical Review Body.

## Author contributions

DN, FC, KV, RBB, RLW, JPB, MH, JWJ, JC, and DJH performed experiments and analyzed data. FC, AT, EMJ, GGL, LH, and DAT ran and analyzed samples for GC-MS. IA analyzed genomic data. DJH and DAT conceived and designed the studies. DJH supervised the studies. DJH, DN, FC, and DAT wrote the paper with input from all authors.

## Supplementary Material

Supplemental data

## Version 1 07/15/2021

In-Press Preview

## Version 2 08/23/2021

Electronic publication
