# Supplementary material for: Prolyl-4-hydroxylase 3 maintains β cell glucose metabolism during fatty acid excess in mice
Source: JCI Insight. 2021 Aug 23;6(16):e140288. doi: 10.1172/jci.insight.140288 (PMC8409982; doi:10.1172/jci.insight.140288)
Supplement: Supplemental data [file jciinsight-6-140288-s247.pdf]

SUPPLEMENTARY INFORMATION

Prolyl-4-hydroxylase 3 maintains β-cell glucose metabolism during fatty acid excess in mice

Daniela Nasteska[1,2,3#], Federica Cuozzo[1,2,3#], Katrina Viloria[1,2,3], Elspeth M. Johnson[4,5], Alpesh Thakker[1,2], Rula Bany Bakar[6], Rebecca L. Westbrook[1,2], Jonathan P. Barlow[7], Monica Hoang[8], Jamie W. Joseph[8], Gareth G. Lavery[1,2], Ildem Akerman[1,2], James Cantley[6,9], Leanne Hodson[4,5], Daniel A. Tennant[1,2*], David J. Hodson[1,2,3*]

[1] Institute of Metabolism and Systems Research (IMSR), University of Birmingham, Birmingham, UK.
[2] Centre for Endocrinology, Diabetes and Metabolism, Birmingham Health Partners, Birmingham, UK.
[3] Centre of Membrane Proteins and Receptors (COMPARE), University of Birmingham, Birmingham, UK.
[4] Oxford Centre for Diabetes, Endocrinology and Metabolism, University of Oxford, Oxford, UK.
[5] Oxford NIHR Biomedical Research Centre, Churchill Hospital, Oxford, UK.
[6] Department of Physiology, Anatomy and Genetics, University of Oxford, Parks Road, Oxford, UK.
[7] Mitochondrial Profiling Centre, School of Sport, Exercise and Rehabilitation Sciences, University of Birmingham, Birmingham, UK.
[8] School of Pharmacy, University of Waterloo, Kitchener, Ontario, Canada.
[9] Division of Systems Medicine, School of Medicine, University of Dundee, Dundee, UK.

[#]These authors contributed equally

*Correspondence should be addressed to: d.tennant@bham.ac.uk or d.hodson@bham.ac.uk

Institute of Metabolism & Systems Research (IMSR), IBR Tower, College of Medical and Dental Sciences, Birmingham B15 2TT, United Kingdom

SUPPLEMENTARY FIGURES

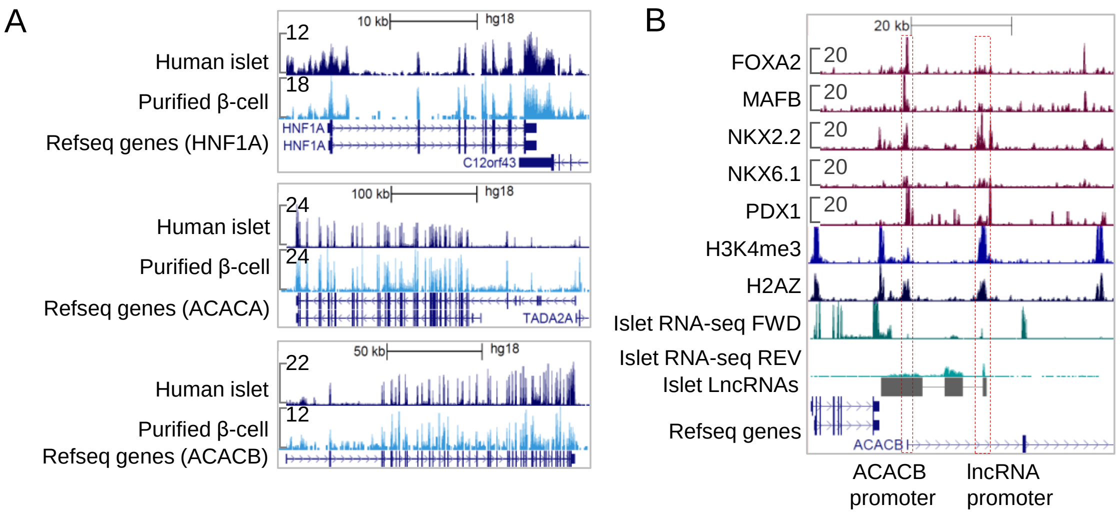

Supplementary figure 1. *ACACB* and *ACACA* gene regulation in human islets. (A) *ACACB* is expressed in human islets and purified β-cells but levels are lower than *ACACA* (B) The *ACACB* promoter is regulated by multiple β-cell transcription factors, with the presence of an antisense-transcribed long non-coding RNA. All data are previously described (1-4), and publicly available via EMBL-EBI and www.isletregulome.com. Data visualization was performed using the open source University of California Santa Cruz (UCSC) Genome Browser (5).

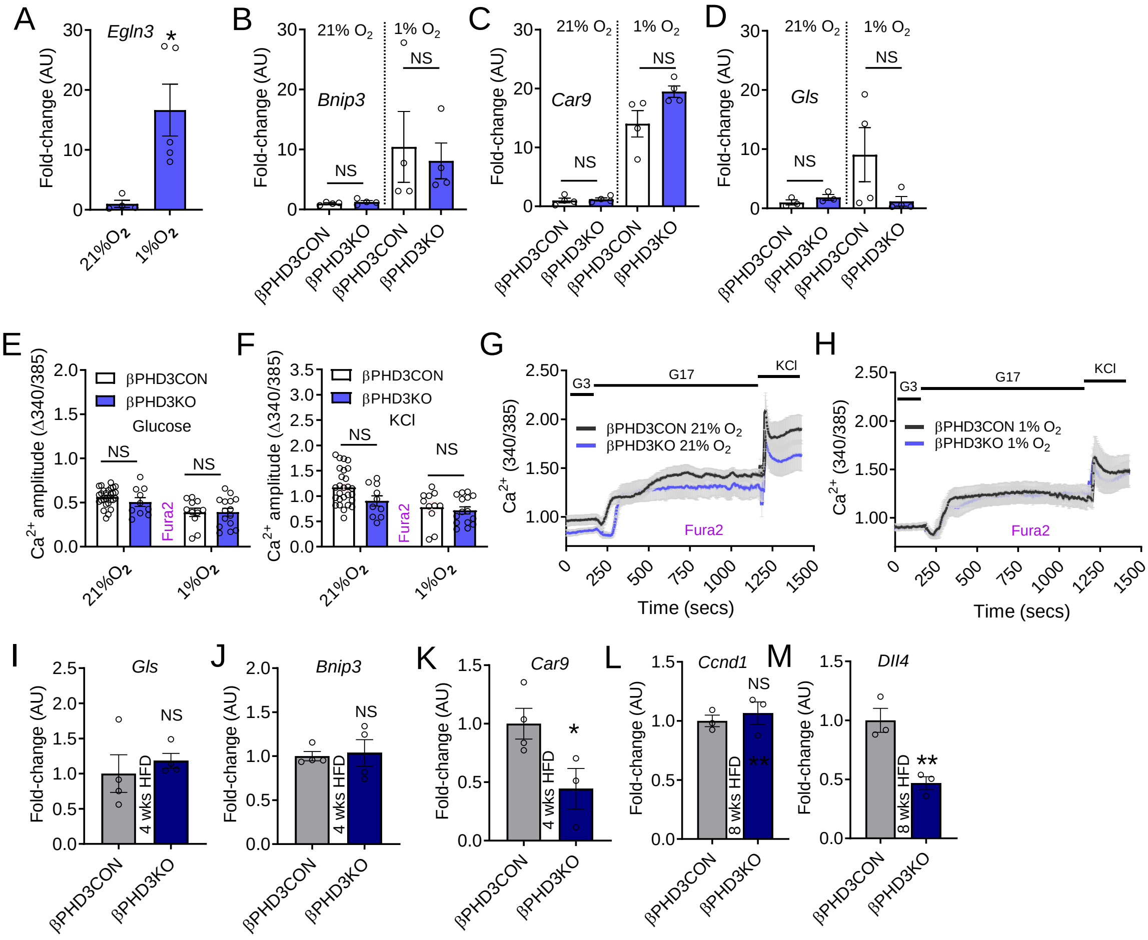

Supplementary figure 2. PHD3 knockout does not induce a hypoxic gene expression or Ca^{2+} signaling phenotype. (A) *Egln3* expression is highly upregulated in wild-type islets following exposure of islets to hypoxic (1% O_2) conditions for 24 hrs (n = 4-5 replicates; 9-11 animals, unpaired t-test). (B-D) Expression of the HIF1α-target genes *Bnip* (B), *Car9* (C) and *Gls* (D), is similar or decreased in βPHD3KO versus βPHD3CON islets exposed to normoxia (21% O_2) or hypoxia (1% O_2) for 24 hrs (n = 4 animals, Kruskal-Wallis test, Dunn's multiple comparison test). (E and F) Glucose- (E) and KCl- (F) stimulated Ca^{2+} fluxes are not significantly different in βPHD3KO versus βPHD3CON islets exposed to normoxia or hypoxia (n = 10-27 islets, 2 animals/genotype, two-way ANOVA; Sidak's multiple comparison test). (G and H) Mean Ca^{2+} traces from βPHD3CON and βPHD3KO islets exposed to (G) normoxia or (H) hypoxia. (I-K) Expression of the HIF1α-target genes *Gls* (I), *Bnip3* (J) and *Car9* (K) is unchanged or decreased in 4 weeks HFD βPHD3KO islets (n = 3-4 animals/genotype, unpaired t-test). (L-M) Expression of the HIF2α-targets (L) *Ccnd1* and (M) *Dll4* is unchanged or downregulated, respectively, in 8 weeks HFD βPHD3KO islets (n = 3 animals/genotype, unpaired t-test). Bar graphs (scatter plot) and line graphs show mean ± SEM. *P<0.05, **P<0.01 and NS, non-significant. PHD3, prolyl-hydroxylase 3.

SUPLLEMENTARY TABLES

Gene name	Forward sequence (5' – 3')	Reverse sequence (5' - 3')
Ppia	AAGACTGAGTGGTTGGATGG	ATGGTGATCTTCTTGCTGGT
Actb	CGAGTCGCGTCCACCC	CATCCATGGCGAACTGGTG
Egln3 (Exon 2)	GCTTGCTATCCAGGAAATGG	GCGTCCCAATTCTTATTCAG
Egn3 (Exon 1)	GGCTGGGCAAATACTATGTCAA	GGTTGTCCACATGGCGAACA
Egln1	TAAACGGCCGAACGAAAGC	GGGTTATCAACGTGACGGACA
Egln2	CATCAATGGGCGCACCA	GATTGTCAACATGCCTCACGTAC
Bnip3	CTGGGTAGAACTGCACTTCAG	CTGGGTAGAACTGCACTTCAG
Car9	GGAGCTACTTCGTCCAGATTCAT	CCGGAACTGAGCCTATCCAAC
Gls	TTCGCCCTCGGAGATCCTAC	CCAAGCTAGGTAACAGACCCT
Ldha	TTCAGCGCGGTTCCGTTAC	CCGGCAACATTCACACCAC
Cpt1a	CTCCGCCTGAGCCATGAAG	CACCAGTGATGATGCCATTCT
Acaca	CTTCCTGACAAACGAGTCTGG	CTGCCGAAACATCTCTGGGA
Acacb	CCTTTGGCAACAAGCAAGGTA	AGTCGTACACATAGGTGGTCC
Pdx1	CCAAAGCTCACGCGTGGA	TGTTTTCCTCGGGTTCCG
Nkx6-1	GCCTGTACCCCCCATCAAG	GTGGGTCTGGTGTGTTTTCTCTT
Mafa	CTTCAGCAAGGAGGAGGTCATC	CGTAGCCGCGGTTCTTGA
Ddit3	CTGGAAGCCTGGTATGAGGAT	CAGGGTCAAGAGTAGTGAAGGT
Xbp1	AGCAGCAAGTGGTGGATTTG	GAGTTTTCTCCCGTAAAAGCTGA
Hspa5	ACTTGGGGACCACCTATTCCT	GTTGCCCTGATCGTTGGCTA
Ccnd1	GCGTACCCTGACACCAATCTC	CTCCTCTTCGCACTTCTGCTC
Dll4	TTCCAGGCAACCTTCTCCGA	ACTGCCGCTATTCTTGTCCC

Supplementary table 1. Primer sequences used for qPCR.

REFERENCES

1. Moran I, Akerman I, van de Bunt M, Xie R, Benazra M, Nammo T, et al. Human beta Cell Transcriptome Analysis Uncovers lncRNAs That Are Tissue-Specific, Dynamically Regulated, and Abnormally Expressed in Type 2 Diabetes. *Cell Metabolism.* 2012;16(4):435-48.
2. Akerman I, Tu Z, Beucher A, Rolando DMY, Sauty-Colace C, Benazra M, et al. Human Pancreatic beta Cell lncRNAs Control Cell-Specific Regulatory Networks. *Cell Metab.* 2017;25(2):400-11.
3. Pasquali L, Gaulton KJ, Rodríguez-Seguí SA, Mularoni L, Miguel-Escalada I, Akerman İ, et al. Pancreatic islet enhancer clusters enriched in type 2 diabetes risk-associated variants. *Nature Genetics.* 2014;46(2):136-43.
4. Akerman I, Maestro MA, De Franco E, Grau V, Flanagan S, Garcia-Hurtado J, et al. Neonatal diabetes mutations disrupt a chromatin pioneering function that activates the human insulin gene. *Cell Rep.* 2021;35(2):108981.
5. Kent WJ, Sugnet CW, Furey TS, Roskin KM, Pringle TH, Zahler AM, et al. The Human Genome Browser at UCSC. *Genome Research.* 2002;12(6):996-1006.
